# Structural Model of RNA Polymerase II Elongation Complex with Complete Transcription Bubble Reveals NTP Entry Routes

**DOI:** 10.1371/journal.pcbi.1004354

**Published:** 2015-07-02

**Authors:** Lu Zhang, Daniel-Adriano Silva, Fátima Pardo-Avila, Dong Wang, Xuhui Huang

**Affiliations:** 1 Department of Chemistry and State Key Laboratory of Molecular Neuroscience, Center for System Biology and Human Health, School of Science and IAS, The Hong Kong University of Science and Technology, Kowloon, Hong Kong; 2 Skaggs School of Pharmacy and Pharmaceutical Sciences, University of California, San Diego, La Jolla, California, United States of America; University of Missouri, UNITED STATES

## Abstract

The RNA polymerase II (Pol II) is a eukaryotic enzyme that catalyzes the synthesis of the messenger RNA using a DNA template. Despite numerous biochemical and biophysical studies, it remains elusive whether the “secondary channel” is the only route for NTP to reach the active site of the enzyme or if the “main channel” could be an alternative. On this regard, crystallographic structures of Pol II have been extremely useful to understand the structural basis of transcription, however, the conformation of the unpaired non-template DNA part of the full transcription bubble (TB) is still unknown. Since diffusion routes of the nucleoside triphosphate (NTP) substrate through the main channel might overlap with the TB region, gaining structural information of the full TB is critical for a complete understanding of Pol II transcription process. In this study, we have built a structural model of Pol II with a complete transcription bubble based on multiple sources of existing structural data and used Molecular Dynamics (MD) simulations together with structural analysis to shed light on NTP entry pathways. Interestingly, we found that although both channels have enough space to allow NTP loading, the percentage of MD conformations containing enough space for NTP loading through the secondary channel is twice higher than that of the main channel. Further energetic study based on MD simulations with NTP loaded in the channels has revealed that the diffusion of the NTP through the main channel is greatly disfavored by electrostatic repulsion between the NTP and the highly negatively charged backbones of nucleotides in the non-template DNA strand. Taken together, our results suggest that the secondary channel is the major route for NTP entry during Pol II transcription.

## Author Summary

In eukaryotic cells, the RNA polymerase II (Pol II) is a central enzyme that reads the genetic information encoded in the DNA template to synthetize a messenger RNA. To perform its function, Pol II needs to have the substrate nucleoside triphosphate (NTP) diffuse into its deeply buried active site. Despite numerous efforts, the NTP entry routes remain elusive: NTP could diffuse only through the secondary channel, or also via the main channel. The structural information of the transcription bubble is essential to study this process, however, the unpaired non-template DNA of the transcription bubble is absent in the available X-ray crystal structures. In this regard, we have built a structural model of the Pol II elongation complex with reconstructed transcription bubble using existing experimental data. We then performed Molecular Dynamics (MD) simulations and applied structural analysis to study the routes of NTP diffusion. We found that sterically the probability of NTP loading through the secondary channel is more than twice that of the main channel. Further analysis of the non-bonded energetic contributions to NTP diffusion suggests that NTP diffusion through the main channel is greatly disfavored by the electrostatic repulsion between the substrate and negatively charged backbones of nucleotides in the non-template strand of the transcription bubble. Altogether, our findings suggest that the secondary channel is the more favorable NTP diffusion route for Pol II transcription elongation.

## Introduction

**Fig 1:**
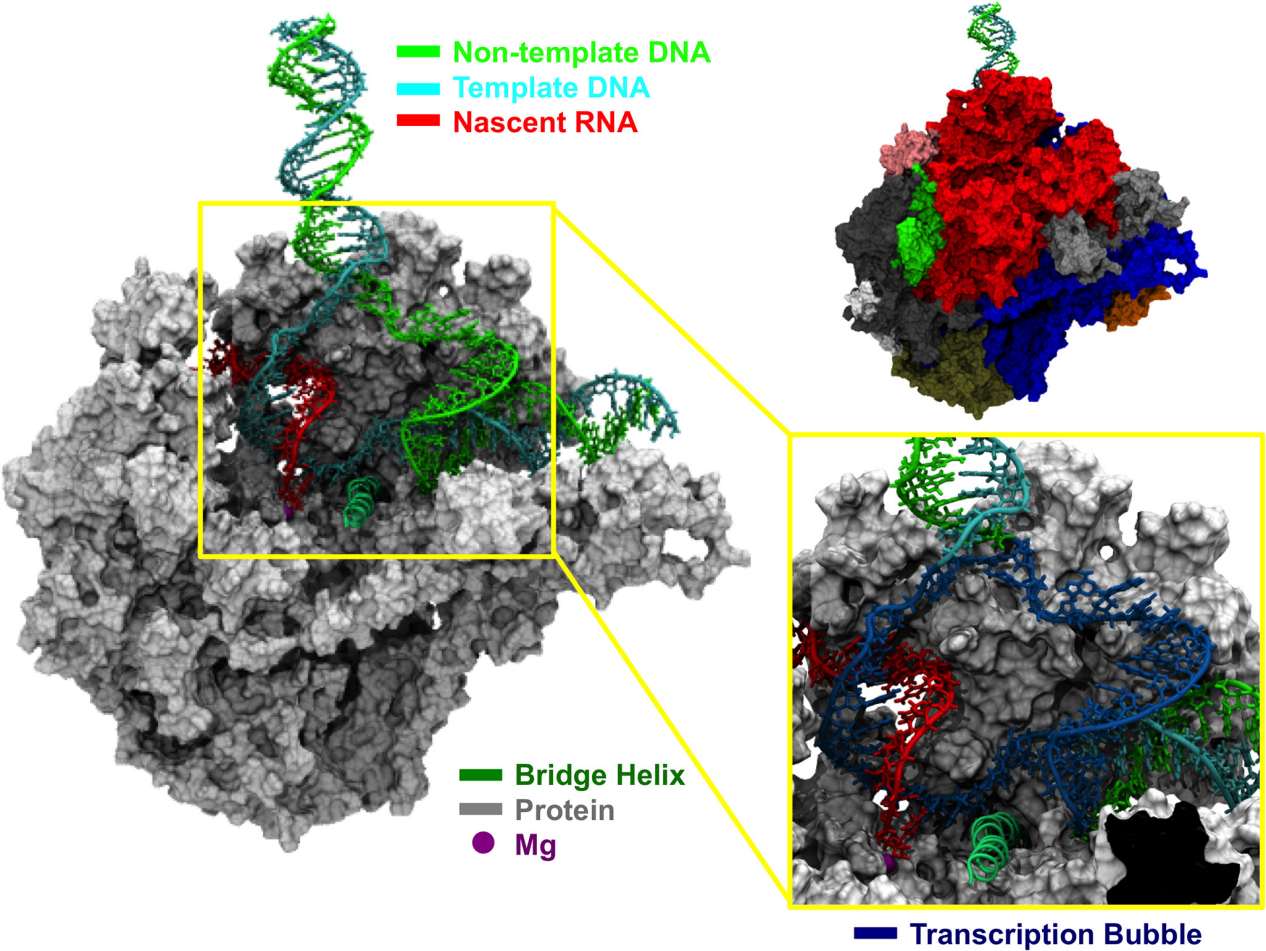
Structural model of Pol II elongation complex with the complete transcription bubble. The left panel shows a cut-view of our Pol II model with a complete transcription bubble. The protein is shown in grey color (surface representation), the bridge helix is shown in dark green color (cartoon representation) and the magnesium ion is shown by a purple sphere. The DNA template, DNA non-template and mRNA strands are shown in cyan, green and red colors, respectively. The right-top panel shows the whole model without the cut view, with the various subunits of Pol II shown in different colors. The right-bottom panel is a close-up of the transcription bubble shown in dark blue.

The RNA polymerase II (Pol II) is a processive eukaryotic enzyme that plays a central role in transcription. It catalyzes the synthesis of messenger RNA (mRNA) with high fidelity and efficiency. Double-stranded DNA (dsDNA) enters the enzyme and unwinds around the active site. The dsDNA strand separates and bends at the downstream edge of the transcription bubble, leaving a DNA strand exposed as template (template DNA strand) for mRNA synthesis [1–5]. Nucleoside triphosphates (NTPs) need to diffuse into the active site for the incorporation to the mRNA strand [6–12]. The nascent mRNA chain remains paired to the DNA template in an RNA:DNA hybrid that extends for about 9 nucleotides [1, 2, 6–9, 13, 14]. Then, the template DNA strand separates from the nascent mRNA strand and re-anneals with the non-template DNA strand at the upstream edge of the transcription bubble (Fig 1).

In the past decades, extensive experimental and computational studies have been performed to elucidate the molecular mechanisms of different steps during the transcription process [1–50]. However, how the NTP diffuses from the surface of the enzyme into the active site is still not fully understood. X-ray crystal structures and biochemical studies have proposed two possible entry routes, namely: secondary channel [7–9, 21, 23, 34, 51–55] and main channel [56–62]. Both NTP loading routes are supported by experimental and computational studies. Crystallographic structures have shown that NTPs can bind to the active site as well as an entry site (E-site) in the secondary channel [7–9, 52], which in turn suggests a two-step mechanism of nucleotide selection. That is, NTP may go through the secondary channel to bind at the E site and then rotate its nitrogenous base to reach the active site. This nucleotide rotation may play an important role in discriminating dNTPs from NTPs, and help to ensure the correct base pairing between the incoming NTP and the template DNA [7, 8]. These ideas have also been confirmed by a computational study, which shows that NTP binding to the E-site in the secondary channel could greatly enhance the NTP binding rate at the active center [21]. Alternatively, NTP is suggested to first bind to a non-catalytic (i+2) site near the downstream edge of the transcription bubble, and then shuttles to the active site via the main channel [56–62]. Even though no crystal structure has been captured with NTP bound to the downstream DNA region, a study using millisecond phase kinetics suggested that the NTP could pre-load to the i+2 site in the main channel prior to translocation into the active site, and this two-step mechanism would increase transcriptional fidelity and efficiency [57]. These observations have been further supported by a recent study using transient state kinetics, which demonstrated that the NTP binding to a non-catalytic template-specific site in the main channel could regulate nucleotide incorporation [59]. However, from a structural point of view, it remains unclear if the main channel contains sufficient space to allow loading of the NTP and how is that a pre-loaded NTP at i+2 site reaches the Pol II active site. In particular, the downstream dsDNA occupies the main channel and leaves little space to allow the diffusion of the incoming NTP [6–9, 13–15, 17, 28, 54, 55]. Moreover, the bridge helix and fork regions seem to sterically block the diffusion route of preloaded i+2 NTP to reach active site along with template base crossover above the bridge helix. In addition, the downstream region of the transcription bubble overlaps with the region proposed to accommodate the incoming NTP, while the transcription bubble is either not included in the crystallographic preparation or its conformation is too flexible to be resolved in the available crystallographic structures of Pol II elongation complex [6–9, 13–15, 17]. Furthermore, the unpaired non-template base in the downstream edge of the transcription bubble might re-anneal with i+2 DNA and over-compete with free NTP. Lacking of full bubble structure makes it difficult to examine the possibility of NTP loading through the main channel based on available elongation structures. A structural model for Pol II elongation complex with the complete transcription bubble is thus crucial for the investigation of the NTP loading routes.

In this work, we have built a structural model of Pol II elongation complex with a complete transcription bubble based on available structures [7–9, 20, 35] (Fig 1). The final model contains 47 nucleotides in both the template and non-template DNA strands and the nascent mRNA strand has been extended to 18 nucleotides in length (Fig 1). Furthermore, we used this model to perform molecular dynamics (MD) simulations to study the effect of the dynamic Pol II complex on the diffusion of NTP. In general, we found that nucleotides that are observed in crystal structures [6–9, 13–15, 17] show less flexibility compared to those for which the electronic density is absent. Based on the stability of base pairing observed in our MD simulations, we identified that the size of the transcription bubble is of 15 nucleotides. To pinpoint possible NTP loading pathways, we analyzed ~1,000 MD snapshots by representing NTP as a sphere. We observed that both channels are possible NTP entry routes, while the number of conformations containing enough space for NTP loading through the secondary channel is more than twice that of the main channel. Based on these results, we constructed a series of structural models with full atomic representation of an NTP molecule located at various positions along the identified diffusion pathways, and performed MD simulations in the presence of explicit solvent. Strikingly, we found that NTPs in the main channel have experienced significantly unfavorable electrostatic interactions compared to those in the secondary channel, mainly due to the strong repulsion from the negatively charged backbones of the nearby nucleotides. Our findings suggest that secondary channel is the major route for the NTP entry during Pol II elongation.

## Results

### Structural model validation by molecular dynamics simulations

We built a structural model of Pol II with complete transcription bubble by extending the DNA (template/non-template) strands and nascent mRNA strand to 47 and 18 nucleotides in length, respectively (see S1 Text.pdb). This structural model equilibrates after 15ns in the MD simulations (S1 Fig). The root mean square deviation (RMSD) values of Cα atoms reach 3Å in the first 10ns. Afterwards, the RMSD increases only slightly and stays around 3.5Å in the last 5ns of the 20ns simulations.

**Fig 2:**
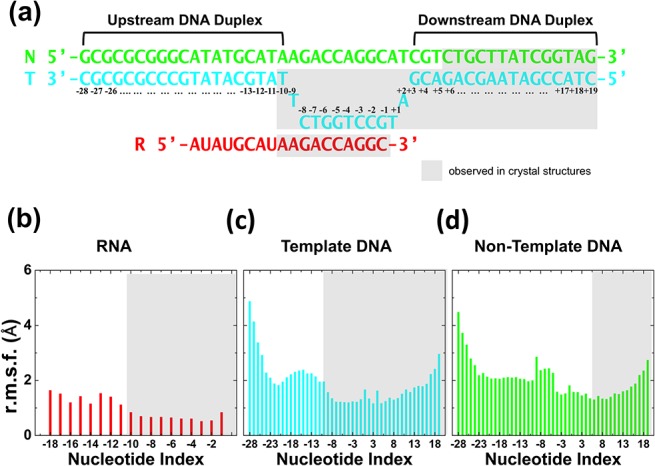
Structural validation of the model by MD simulations: Nucleic acids flexibility. (a) The scheme represents the nucleic acid scaffold used in our simulation (numbers denote nucleotide positions). (b)-(d) The RMSF, per nucleotide position, observed in our MD simulations for: mRNA, template DNA and non-template DNA. Grey background denotes those nucleotides that have been observed in crystallographic structures.

Nucleotides located in various regions of Pol II show different flexibility in MD simulations (Fig 2). The nucleotides of the nascent mRNA strand from positions i-1 to i-10 demonstrate small mobility, with the root mean square fluctuation (RMSF) < 1.0Å (Fig 2B). The mRNA beyond position i-11 is more exposed to the solvent and in consequence exhibits a higher flexibility (Figs 1 and 2B). This is consistent with missing electron density in crystallographic structures [6, 9, 14]. The template DNA strand contains 47 nucleotides and those located in the RNA:DNA hybrid region (from positions i-1 to i-8) show small fluctuations in the MD simulations (Fig 2C). The nucleotides from positions i+5 to i+16 are stable with an RMSF < 2.0Å as they are paired with the nucleotides of non-template DNA strand under a constrained protein environment. The non-template strand separates from the template DNA strand near the bridge helix in the downstream edge, and re-anneals with the template strand in the upstream edge of the transcription bubble. The nucleotides of the non-template strand located near or within the bubble region (from i-9 to i+4) do not have stable base pairing partners, thus fluctuate significantly in the simulations (Fig 2D). On the contrary, the non-template nucleotides in the downstream dsDNA show less fluctuation (Fig 2D). Furthermore, terminal nucleotides of both DNA strands present higher fluctuations (Figs 2C and 2D). Especially for the region between i-24 to i-28 of the upstream DNA, the RMSF could be as high as 4.5Å (Figs 2C and 2D). Comparison to crystal structures indicates that the structural components observed in the X-ray crystals [6–9, 13–15, 17] are relatively stable in the MD simulations, while those absent or disordered in the crystal structures show more significant fluctuations.

**Fig 3:**
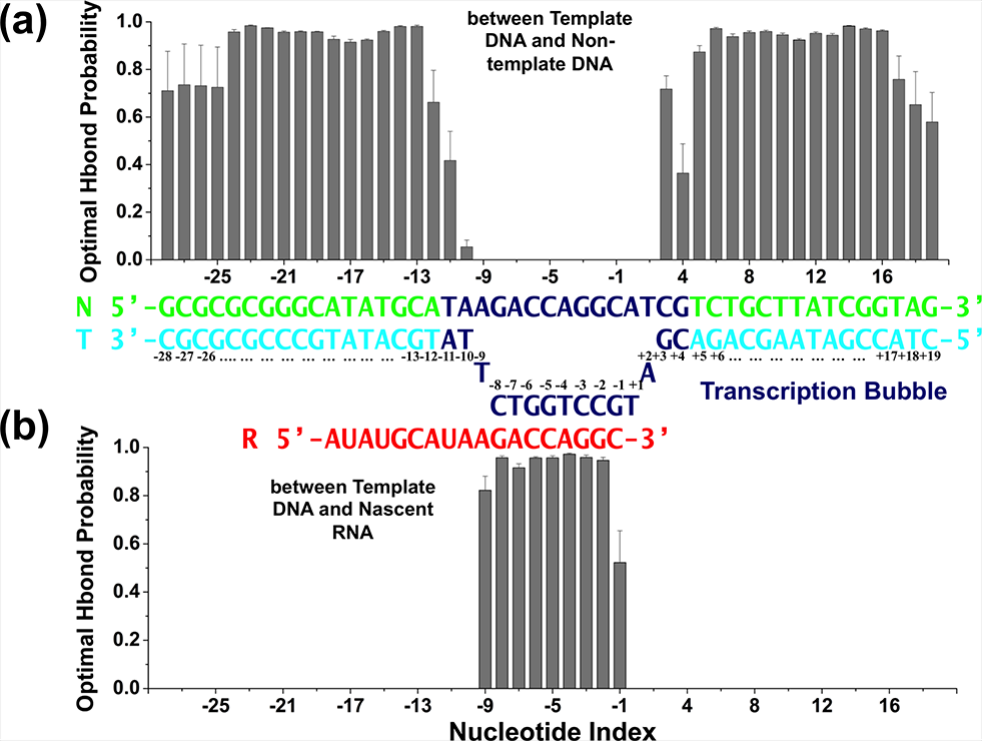
Structural validation of the model by MD simulations: Base-pairing stability. The bar plots show the probability of finding the optimal number of hydrogen bonds for: (a) between the template and non-template DNA strands, and (b) between the DNA-RNA hybrid. In the middle, the nucleic acid scaffold scheme is demonstrated with the region where the DNA duplex opens to form the transcription bubble shown in dark blue.

By measuring the base pairing stability in MD simulations we found that the transcription bubble contains 15 nucleotides (Fig 3). The nucleotides from positions i-24 to i-12 region of the upstream DNA strands and from i+5 to i+16 of the downstream duplex show stable base pairing with hydrogen bonds probability > 60% (Fig 3A). The dsDNA starts to unwind (probability < 60%) at i+4 and reunites at i-12 site. Furthermore, nucleotides in the RNA:DNA hybrid from i-2 to i-9 are well paired, with hydrogen bonds probability > 70% (Fig 3B). The base pairing stability for the 3’-terminus nucleotide of the mRNA (i-1) is slightly reduced (with a hydrogen bond probability of ~50%) due to its higher flexibility compared to the nucleotides locating from i-2 to i-9 (Figs 2B and 3B). The base pairing between the nucleotides of the hybrid is completely lost (with a probability dropping to zero) starting from i-10, indicating that the template DNA and nascent mRNA strands separate at position i-10 (Fig 3B).

### Sterically, the secondary channel shows higher probability than the main channel for NTP loading

**Fig 4:**
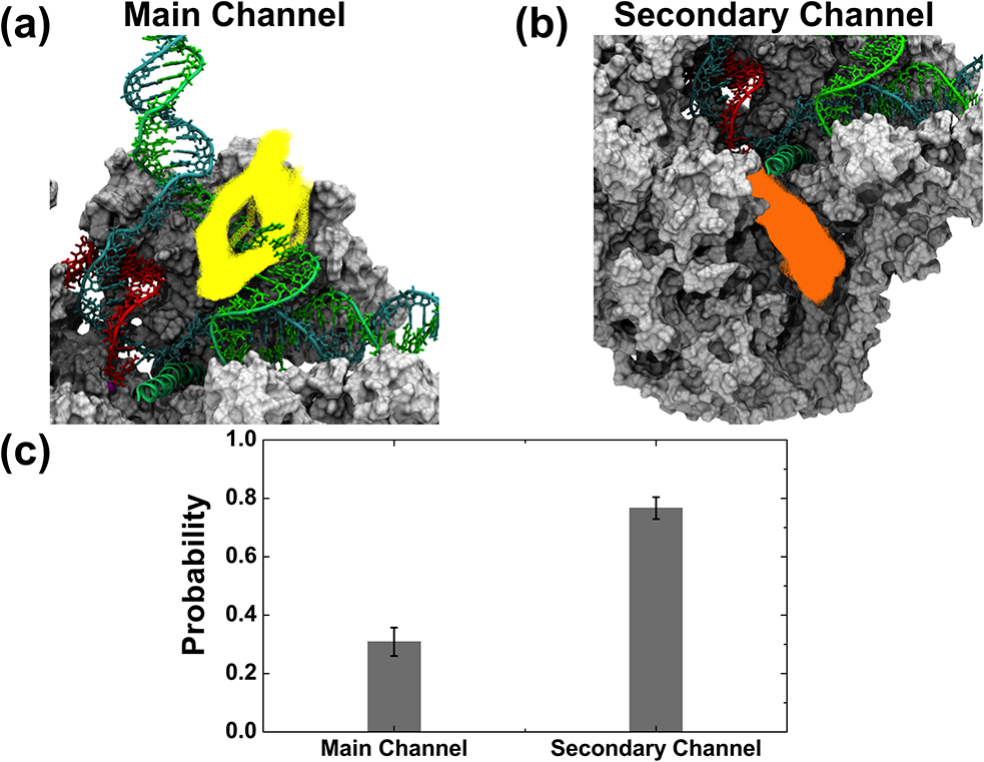
The secondary channel exhibits higher probability for NTP entry than the main channel. The cavity analysis was performed using the program CAVER (see Methods) with an ensemble of MD conformations of the elongation complex (without NTP). (a) The most possible pathways with enough space to allow the pre-loading of a NTP to the i+2 site are shown in yellow. Similarly, (b) shows the most possible pathways for a NTP to reach the active site in orange. In (c), the bar plot shows the probability of finding MD conformations with enough space for NTP diffusion through the main channel versus the secondary channel (see Methods). The template DNA (cyan), non-template DNA (green) and RNA (red) strands are shown with tube and licorice representations. The protein components are shown in grey with a cut view with a surface representation.

Using the program CAVER [63], we analyzed the MD conformational ensemble to find pathways that could allow the diffusion of a NTP molecule into the active site region of Pol II (see Methods). We discovered potential diffusion pathways through both the main channel and secondary channel. For the main channel, we identified a bifurcated pathway that connects the Pol II surface to the i+2 binding site (Fig 4A). Different from previous models for the NTP entry through the main channel [56–62], neither branch of the pathway is located along the downstream DNA duplex, indicating that the available space in the downstream region is too limited to allow the passage of NTP. However, as shown in Fig 4A, the NTP may diffuse from the solvent to the i+2 binding site through the bifurcated pathway located at both sides of the non-template DNA strand in the transcription bubble region. In the later part of this article, we refer to this branched pathway as the main channel. We also discovered a pathway that can lead directly from the enzyme surface to the i+1 active site (Fig 4B). This pathway goes through the funnel and pore region, and is consistent with the previously proposed secondary channel, Hence we refer to this pathway as the secondary channel.

The probability of finding pathways for NTP (simulated as a hard sphere with a radius of 3.5 Å) to the i+1 active site along the secondary channel is more than twice that of finding the pathways to the i+2 binding site along the main channel (Fig 4C). In particular, the main channel pathway was only observed in ~31% of the MD conformations, while almost 77% of the MD conformations contain enough space for NTPs to diffuse to the active site through the secondary channel. Thus, though both channels contain enough space for the loading of NTP, the higher probability of finding pathways to the active site makes the secondary channel the major NTP entry route.

**Fig 5:**
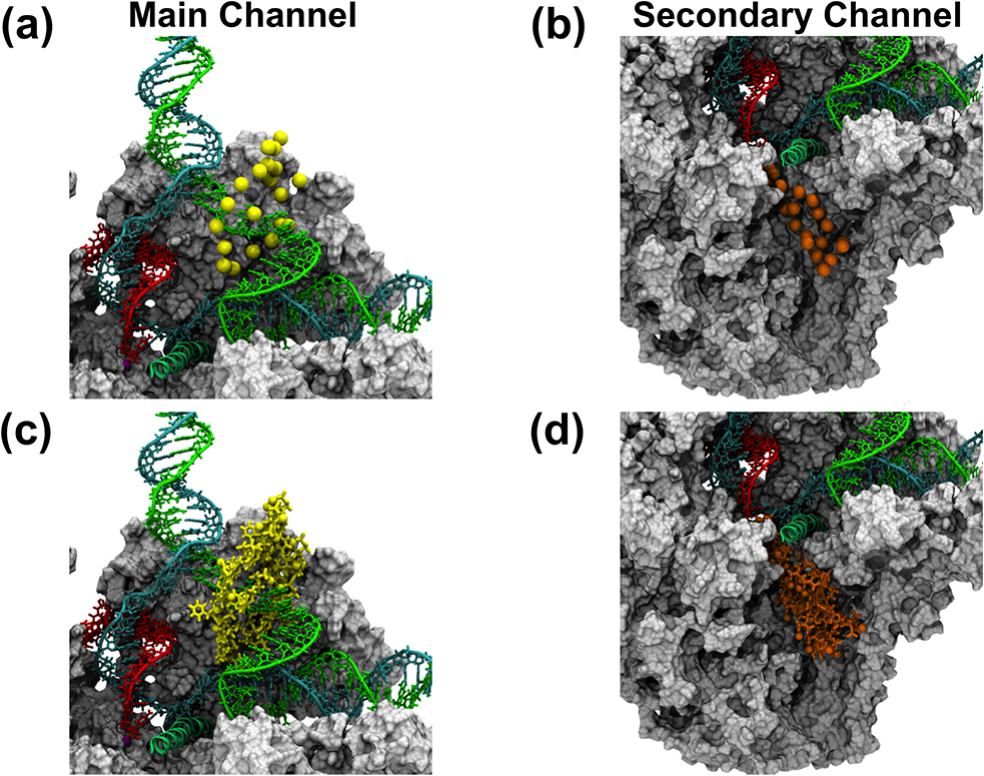
MD relaxation of NTP in the main channel and the secondary channel. (a-b) Initially, the points constituting the entry routes through the main and secondary channels were divided into 20 clusters. The yellow and orange spheres represent the center of each cluster for the main channel and the secondary channel, respectively. (c-d) A NTP molecule (in licorice presentation) accompanied by a bound magnesium atom (sphere representation) was aligned to each of the cluster centers (shown in (a-b)) by its center of mass (see Methods). These conformations were used as starting points for relaxation via MD simulations (see Methods). The template DNA, non-template DNA and mRNA strands are shown in cyan, green and red, respectively, with tube and licorice representations. The cut-view of the protein is shown in grey with a surface representation.

In order to consider the atomic structure of NTP molecules rather than simply treating them as spheres, we further modeled the all-atom NTP conformations at various locations along the above-mentioned loading pathways (Fig 5). In particular, we superimposed the center of mass (c.o.m.) of NTP with the locations of spheres identified by CAVER [63] and performed energy minimization. In this way, the NTPs could fit themselves into the space along the pathways (see Methods). We observed again that the secondary channel was still more favorable than the main channel. We found that the NTP molecules located along the main channel pathway deviated more from their initial positions compared to those along the secondary channel (S2 Fig). In particular, ~50% of the NTP molecules in the main channel move their c.o.m. > 1.0Å away from the initial locations after energy minimization. Three of them even show deviations > 2.0Å, with the highest value being of 3.5Å (S2A Fig). Besides, the shortest atomic distance deviation of NTP molecules in some locations of the main channel is as high as 2.0Å (S2A Fig). In contrast, NTP molecules in the secondary channel do not need to move significantly from their starting positions, where 90% of the conformations show an atomic distance deviation < 1.0Å (S2B Fig). By taking into account the steric effects of the NTP molecules along the pathways, the comparison of the NTP molecules’ positional deviations along the two channels after energy minimization also supports that the secondary channel is the most favorable route for the NTP entry.

### NTP diffusion through the secondary channel is energetically more favorable than the main channel

**Fig 6:**
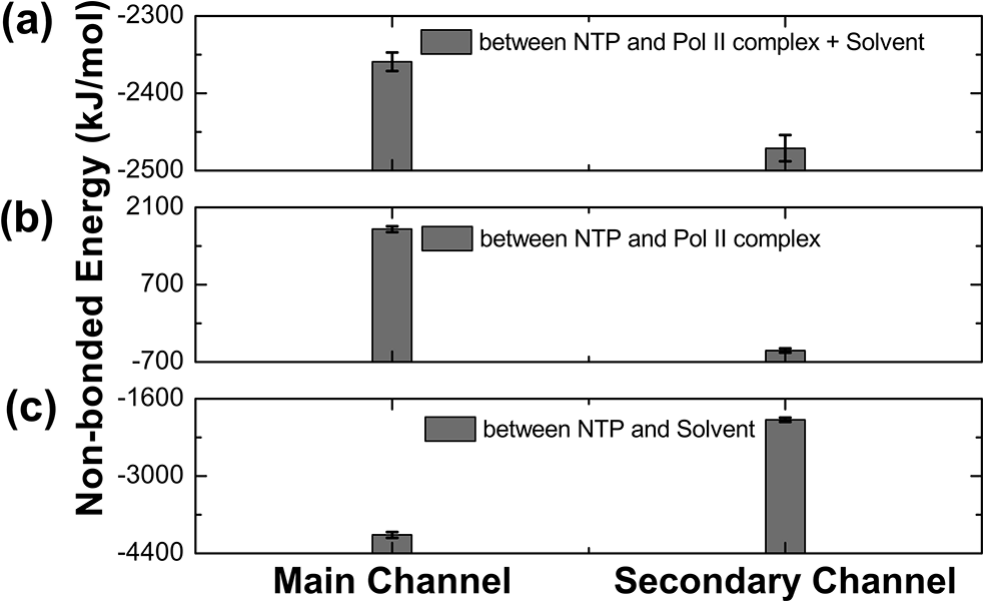
Non-bonded interactions NTP experiences revealed by MD. The plots compare the energetic contribution of non-bonded interactions (electrostatic + vdW) along the main channel and secondary channel for: (a) NTP and its environment (the Pol II complex and solvent); (b) NTP and the Pol II complex; and (c) NTP and solvent. In these calculations, the Pol II complex includes the protein and nucleotides. The solvent contains water molecules together with the counter-ions. The results shown in (a) are the total contributions from the Pol II complex (shown in (b)) and the solvent (shown in (c)) (see Methods).

To determine the stability of NTP along the pathways under the dynamic protein environment, we performed MD simulations starting from the energy minimized conformations and found that the NTP molecules have more favorable non-bonded interactions with the environment in the secondary channel than in the main channel (Fig 6A). Two components contribute to this energy difference: NTPs interacting with Pol II (protein and nucleotides) and the solvent (water and counter ions) (Figs 6B and 6C). We also find that the non-bonded energy difference is mainly due to the discrepancy of the Coulomb interaction (S3A Fig). Analysis of electrostatic potential shows a negatively charged potential along the main channel, while the secondary channel shows a surface with a nearly even distribution of positive and negative electrostatic potential (S4 Fig), suggesting a more favorable pathway for NTP diffusion through the secondary channel. The van der Waals (vdW) interactions (represented by the LJ interactions) do not show a significant difference between the two channels (S3B Fig).

**Fig 7:**
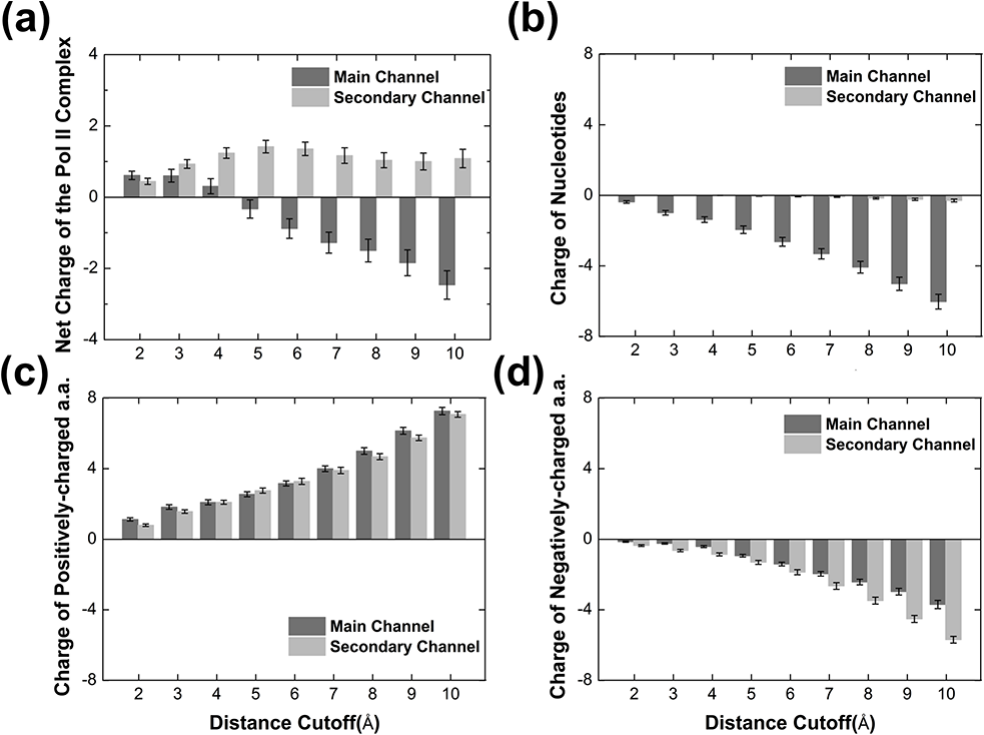
Distribution of charges surrounding the NTP during MD simulations. (a) The plot shows the net charge of the Pol II (protein and nucleotides, y-axis) within certain radius (x-axis) of the NTP in the diffusion pathways through the main channel (dark grey) or the secondary channel (light grey). (b)-(d) The same as in (a) but for the charges of the nucleotides, positively-charged amino acids (a.a.) and negatively-charged a.a. around NTP, respectively.

The NTP has more stable non-bonded interactions with the Pol II complex in the secondary channel than along the main channel. As shown in Fig 6B, the interaction between NTP and the Pol II complex in the main channel is quite unstable. This could be explained by the fact that in the main channel the charges surrounding the NTP are mainly negative (Fig 7A), thus destabilizing the also negatively charged NTP. In contrast, the NTP has more stable interactions with the Pol II complex in the secondary channel (Fig 6B), because the overall charge distribution surrounding the NTP is mainly positive, hence a favorable electrostatic environment for the NTP (Fig 7A). Further analysis shows that the charge difference between two channels arises from the nucleotides (Fig 7B) while the charge distributions of the amino acids surrounding NTP are similar along both channels (Figs 7C and 7D). As shown in Fig 4A, the main channel bifurcated pathway locates near to the negatively charged phosphate backbone of the non-template DNA strand, therefore the NTP which also carries negative charge would be destabilized in the main channel by the repulsion from the nearby phosphate backbones. On the contrary, the pathway of the secondary channel is distant from the nucleotide-dense regions, resulting in more stable interaction between NTP the Pol II complex (Figs 4B and 7B).

The analysis of the interaction between NTP and solvent demonstrates an opposite pattern: NTPs in the main channel are more stabilized by the solvent than those in the secondary channel (Fig 6C). This may be related to a higher exposure of the main channel to the solvent and thus the screening effect due to the counter ions and solvent molecules helps to stabilize NTPs (e.g. ~2 Na^+^ within 10Å of NTP by average). On the contrary, the secondary channel is more deeply buried in the protein, thus NTPs along this pathway are poorly shielded by counter ions and solvent molecules (e.g. around half of MD conformations containing no Na^+^ within 10Å of NTP), leading to a less stable interaction between NTPs and solvent.

In summary, even though solvent screening effect helps to stabilize NTPs in the main channel, NTPs still prefer the secondary channel if we consider the nucleotide repulsions along the main channel during MD simulations. This also supports that the secondary channel is the major NTP entry route.

## Discussion

Although previous studies have provided great insight into the NTP loading process during Pol II transcription, it is still unclear how the NTP diffuses into the active site from the surface of the enzyme. Two routes have been proposed: a secondary channel directly leading from the enzyme surface to the active site [7–9, 21, 23, 34, 51–55] and a main channel following the downstream DNA duplex binding region into the i+2 binding site [56–62]. Until now, crystal structures have been able to capture NTP binding only to the secondary channel [7–9, 52]. Furthermore, based on the available structural information [6–9, 13–15, 17, 28, 54, 55], it is unclear if the downstream DNA in the main channel would impede the passage of NTP into the i+2 site. However, biochemical studies have proposed that NTPs could diffuse through the main channel and bind to a non-catalytic site to accelerate RNA synthesis [57, 59]. Even so, the role of main channel still remains controversial, as the non-template DNA part of the transcription bubble overlapping with the main channel is crucial for the study of NTP diffusion routes while its conformation is absent in the available crystal structures of Pol II elongation complex [6–9, 13–15, 17].

In this work, we have built a structural model of Pol II with full transcription bubble (Fig 1). Based on this model, we performed MD simulations and used an ensemble of MD snapshots to study the NTP entry routes. Firstly, the NTP was treated as a sphere and MD conformations were examined to find pathways with enough space to allow NTP diffusion. Consistent with the previously proposed model for NTP loading through the secondary channel, our analysis also identified the same NTP entry pathway [7–9, 21, 23, 34, 51–55] (Fig 4B). In contrast, different from the previously hypothesized main channel pathway along the Pol II downstream DNA duplex [56–62], we found an bifurcated alternative pathway from the enzyme surface to the i+2 non-catalytic binding site, locating at two sides of the unpaired non-template DNA strand in the bubble region (Fig 4A). Furthermore, though analysis of the MD conformational ensemble shows that both channels are possible routes for NTP entry by considering only the steric effect, the probability of finding the secondary channel is more than two times higher than that of the main channel (Fig 4C).

Finally, to take into account the atomic details of the NTP molecule, we constructed all-atom models with NTP molecules at various locations along the pathways (Figs 5C and 5D) and further performed MD simulations to examine their stability. Strikingly, we discovered that NTPs in the secondary channel are energetically much more stable than those in the main channel (Fig 6A). In particular, NTP molecules experience significantly more favorable electrostatic interactions with the Pol II complex in the secondary channel than in the main channel (Fig 6B), mainly because the NTP is repelled by the nearby negatively charged phosphate backbone of the non-template DNA in the main channel (Fig 7B). In addition, investigation of the non-bonded interaction between NTP and solvent suggests that the screening effect from the counter-ions and solvent helps to stabilize the NTPs in the main channel (Fig 6C), as the bifurcated pathway is shorter and more exposed to the solvent than the secondary channel. Nevertheless, after taking into account of the overall environment including Pol II and solvent, we found that the NTP is still greatly favored energetically when entering through the secondary channel compared to the main channel. Our findings are consistent with the previous proposal that the secondary channel is the major pathway for NTP entry [7–9, 21, 23, 34, 51–55].

Our current study provides a qualitative comparison of the energetics for NTP to diffuse via the two channels; however, the actual energy difference between the two channels might be not accurate due to simplicity of the energy calculations that we have used. In this regard, free energy calculations may help to investigate an accurate value for the potential of mean force of NTP diffusion through the channels. Nevertheless, due to the complexity of this system, directly performing free energy calculations is challenging and involves enormous computational cost, since it requires sampling all the relevant conformations of the NTP along the diffusion pathways, including not only the conformations of NTP molecule itself, but also its distribution along the pathways and any associated conformational change of Pol II.

Based on our modeling results, we can predict key residues in the NTP diffusion that can be tested by experiment. For example, due to the more limited space for NTP loading through the main channel, we expect that mutations of alanine or glycine along the main channel (for example, A75 in chain B and G178 in chain A) to bulky amino acids or analogs could further reduce the probability for NTP diffusion through the main channel. Since electrostatic interactions play an important role for NTP diffusion, mutations of positively-charged amino acids (for example, K176 in chain A, K422 in chain B and R249 in chain B) to the negatively-charged amino acids could cause stronger repulsion to the NTP in the main channel, thus further impede the NTP entry through the main channel.

In vivo, RNAP may associate with various transcription factors and the presence of these proteins may affect the diffusion of NTP. For example, the elongation factor Spt4/5 [33, 35] locates near the entrance of the main channel pathway. To examine its effect on NTP diffusion, we performed channel analysis and MD simulations of the Spt4/5-Pol II complex (see S2 Text for modeling details). We found that Spt4/5 could further reduce the probability of NTP diffusion through the main channel (see S2 Text and S5 Fig). We are aware that the structure of Spt4/5 that we adopted [35] is not full-length. Therefore, there exist possibilities that the main channel pathway may be further blocked with the presence of full-length Spt4/5. During the proofreading process, the transcription factor TFIIS could bind to the secondary channel of Pol II to cleave the mis-incorporated RNA nucleotides. Its presence will occupy the entire secondary channel [17], and thus exclude the possibility for the NTP to diffuse to the active site through the secondary channel.

In future studies, it will be of interest to consider the coupling of NTP diffusion, DNA:RNA oscillation and translocation. According to the Brownian-ratchet model, the translocation motion is coupled to NTP binding, therefore it exists the possibility that RNAP could oscillate between the pre- and post-translocation states during NTP loading. However, in the current work each individual MD simulation is limited to the length of 20ns, and within this short time-scale it is not possible to observe the oscillation of the DNA/RNA hybrid between pre- and post-translocation states (Previous work suggests the timescale for the oscillation in the absence of NTP is at tens of microseconds [20]). The mechanism of the NTP shuttling from the i+2 site to the active site has been suggested by a few previous studies. For example, the kinetic studies by Kennedy and Erie suggested that the NTP could diffuse through the main channel, bind to the i+2 site and shuttle to the active site along with translocation [59]. Gong *et al*. also found that there is enough space for the NTP shuttling from the i+2 site to the active site based on a minimum scaffold of transcriptional complex [57]. The NTP shuttling to the active site is a plausible model, but there also exists other alternative mechanisms. For example, it is possible that the interaction of the NTP at the i+2 site simply has allosteric regulatory role of translocation, downstream bubble opening or catalysis. Our current work is focused on determining the possibility for NTP to diffuse into Pol II via the main channel (to the i+2 site) or the secondary channel (to the i+1 site, i.e. active site). For this purpose, we chose to use the post-translocation state of Pol II, this is because both sites (i+1 and i+2) are available for NTP binding, while in the pre-translocation state only the i+2 binding site is available.

In conclusion, our work has provided insight into the possible routes of NTP entry by performing unbiased pathway searching along both the main channel and secondary channel using an ensemble of MD conformations. Two proposed entry channels are compared sterically and energetically. Our results have shown that the main channel is still a possible route by solely considering the steric effect. However, if we consider the substantial energy difference between the two channels, the secondary channel becomes the favorable route for NTP diffusion. Our work also lays foundation for future studies of the kinetics and thermodynamics of NTP loading in Pol II.

## Methods

### Structural model construction for the complete transcription bubble

The structural model of Pol II with full transcription bubble was built based on available structures [7–9, 20, 35] (see S1 Text). The post-translocation state model built previously for the translocation study [20] was used as our initial conformation. The downstream DNA was extended by 4 nucleotides after aligning to the crystal structure (PDBID: 2E2H [7]) with the P and O5’ atoms of the nucleotides in positions from i+1 to i+6 of the template strand (refer to the nucleotides positions in Fig 2A). Afterwards, the all-atomic model of eukaryotic RNAP II elongation complex containing full transcription bubble with Spt4/5 bound to the clamp domain [35] (quoted as “Pol II-Spt4/5” later in the text) was used as reference to build the transcription bubble and the upstream DNA. We noticed that there exist other models that may serve as alternative starting structures for our modeling such as the archaeal RNAP elongation complex model with the full transcription bubble [33]. Specifically, we aligned Pol II-Spt4/5 structure to our model with longer downstream DNA by P, O5’ and C5’ atoms of the nucleotides at i+1 ~ i+3 positions. To model the downstream edge of the full transcription bubble, we used the coordinates of the nucleotides at positions i+3 to i+6 of the aligned Pol II-Spt4/5 to replace the corresponding nucleotides in our model. Furthermore, the fragments of template DNA starting from i-10 to i-28 of the 3’-end and the non-template DNA strand from the 5’-end to i+7 were also extracted from the aligned Pol II-Spt4/5 and inserted into our model. Because the nucleotides were disconnected around the upstream edge of the transcription bubble due to the structural alignment, we applied energy minimization solely for nucleotides located at i-8 ~ i-10 to connect them. One extra nucleotide pair was appended to the downstream dsDNA terminal by aligning the nucleotides’ atoms C5 and P from positions i+10 ~ i+14 of the Pol II-Spt4/5 to our model. The nascent mRNA strand was also extended: we aligned Pol II-Spt4/5 to our model by the carbon atoms of nucleotides bases at positions from i-6 to i-9; then the fragments of the aligned Pol II-Spt4/5 from i-9 to i-18 were inserted into our model. The final sequences shown in Fig 2A were achieved by point mutations of the nucleotides using the molecular modeling suite Coot [64].

We also manually fixed some structural clashes between the amino acids and the nucleotides. First, we aligned Pol II-Spt4/5 to our model by the Cα atoms of Rpb1 residues 200~240 and 270~310. Rpb1 residues 248~260 from the aligned Pol II-Spt4/5 were extracted and used to replace the corresponding amino acids in our model. PHE with residue ID 252 was rotated to avoid its positional overlap with the nearby nucleotide. To correct the clash between the amino acids and the nucleotides in the 5’ exit of nascent mRNA strand, we aligned Pol II-Spt4/5 to our model by the phosphate atoms of nucleotides from i-10 to i-19 and then extracted the Rpb1 residues 60~65 from the aligned Pol II-Spt4/5 to replace these six residues in our model. Similar modifications were also made to the Pol II Rpb2. In particular, we aligned Pol II-Spt4/5 to our model by the Cα atoms of Rpb2 residues 480–530. Afterwards, the fork loop 4 (Rpb2 residues 501–510) from the aligned Pol II-Spt4/5 was extracted to replace the corresponding residues in our model. The side chain of Rpb2 residue 430 was rotated and residues 436~447 and 918~934 were also manually pulled to avoid their clash with the nucleotides. The final structural model contains template/non-template DNA strands and mRNA strand of 47 and 18 nucleotides in length, respectively (Figs 1 and 2A).

### MD simulations of Pol II elongation complex

The amber99sb force field [65] with modifications on nucleotides [66–71] was used to perform all-atom MD simulations. To maintain the coordination between the zinc and the protein, we have added harmonic restraints with a force constant of 2261.03 kJ.mol^-1^.Å^-2^ between zinc ions and their coordinated cysteine residues. The protonation states of the histidines in our model were assigned as previously described [20].

The structural model with the complete transcription bubble was used as our starting conformation. To remove the steric clashes, we first performed a 5,000-steps energy minimization with the steepest descent algorithm by freezing the nucleotides. Furthermore, another 50,000 steps energy minimization was performed for the Pol II system in vacuum to smooth the contact between the amino acids and nucleotides. Next, we solvated the whole system in a water box of 160Å*188Å*160Å (α = 90°, β = 90°, γ = 90°) and neutralized it by adding 141 sodium ions. Our simulation system contains 481,887 atoms in total, including 139,789 TIP3P water molecules [72]. Afterwards, 10,000 steps energy minimization was performed for the whole system. To further relax the Pol II in solvent, position restraints with a force constant of 10 kJ.mol^-1^.Å^-2^ was enforced on all the heavy atoms of the Pol II complex and simulation was performed for the whole system for 200ps under an NVT ensemble (T = 310K). The final configuration from the position-restrained simulation was used to initiate 4 independent NVT production (T = 310K) simulations with different initial velocities. The first 500ps were used for temperature annealing from 50K to 310K, followed up by 20ns simulations with the temperature kept at 310K. We stored snapshots every 20ps. The long-range electrostatic interactions beyond the cut-off at 12Å were treated with the Particle-Mesh Ewald (PME) method [73]. The Lennard-Jones interactions were smoothly switched off from 10Å to 11Å. The neighbors list was updated every 10 steps. An integration time step of 2.0ps was used and the LINCS algorithm [74] was applied to constrain all the bonds. All the MD simulations were performed using Gromacs 4.5 [75]. A PDB of the energy-minimized structure is available in the SI as S1 Text.pdb.

### Structural validation

As shown in S1 Fig, the system becomes equilibrated after 15ns. Thus the conformations from the last 5ns of MD simulations were adopted for the structural validations. To investigate the flexibility of the nucleotides, we used the C4 atom of each nucleotide for RMSF calculations for each trajectory. Afterwards, we averaged the data from all the trajectories and the resulting RMSF values for mRNA, template and non-template DNA are shown in Figs 2B–2D. To investigate the base pairing stability, we calculated the probability of optimal hydrogen bonds formation during the MD simulations (Fig 3). In particular, 2 and 3 hydrogen bonds are defined as the “optimal number of hydrogen bonds” for A-T(U) and C-G pairs, respectively. We applied the default hydrogen bond definition in Gromacs [75] (3.5Å for distance cut-off of donor-acceptor and 30° for angle cut-off of acceptor-donor-hydrogen) to determine the number of hydrogen bonds observed in MD simulations. Then we calculated the percentage of snapshots showing the optimal hydrogen bond number for each trajectory. We applied the bootstrapping algorithm with replacement to get the average hydrogen bond probability (10 iterations with 10 random trajectories per iteration). We also validated the protonation states of titratable residues and discussed the placement of counter ions in our MD simulation model (S6 and S7 Figs and S1–S6 Tables).

### Channel analysis based on MD conformations

The last 5ns of MD simulations (1,004 MD snapshots) were used to analyze the channels. The program CAVER [63] was used to search for cavities that might form pathways connecting the protein surface to the binding site (Figs 4A and 4B). For this purpose, the NTP was simulated as a sphere of radius 3.5Å (as suggested previously in [21]). Default settings were used for other input parameters to search the pathway, except that “shell_radius” and “shell_depth” were set to be 30Å and 40Å considering the distance from the Pol II surface to the buried binding site (see [63] for details). For the main channel, the i+2 on-catalytic binding site was used as the initial point for the pathway search. All the pathways that could allow the sphere to go through were identified and divided into groups according to their mutual geometry distances. These groups are then ranked according to the number and the cost of pathways in each group (see [63] for methodology details). During our analysis, we only focused on the first group, which is the most probable or the one of the highest priority (Fig 4A). We then applied the bootstrapping algorithm to randomly select 100 MD conformations from our ensemble for 100 times, and calculated fraction of these conformations containing pathways that belong to the most probable group (Fig 4C). A similar procedure was applied to search the pathways and calculate the probability for the secondary channel, but using the i+1 active site as the initial point in CAVER (Figs 4B and 4C).

### MD simulations for the NTP loading

We first used the k-centers algorithm in MSMBuilder-1.0 [76] to cluster all the points constituting the pathways into 20 microstates according to their positional similarity. We then extracted the central point of each microstate (Figs 5A and 5B) and used these points as the reference positions for placing NTP molecules in the channels. According to the sequence shown in Fig 2A, the matched NTPs for the main channel and secondary channel are UTP and ATP, respectively. Each NTP molecule carries one magnesium ion coordinated with its P_α_, P_β_ and P_γ_ atoms to simulate the Mg B in the crystal structure [7]. The c.o.m. of the NTP with Mg^2+^ was aligned to the microstate center (Figs 5C and 5D). We then used the aligned NTP and the Pol II MD conformation corresponding to the specific center to build up the structural models with NTP along the channels. Because we used 20 microstate centers for each channel, we have 40 different structural models in total, including 20 models with (UTP-Mg)^2+^ in the main channel and other 20 models with (ATP-Mg)^2+^ in the secondary channel.

The parameters of the protein residues, DNA, RNA and the ions were taken from the all-atom amber99sb force field [65] with modifications on nucleotides [66–71]. To simulate NTP, parameter modifications on the polyphosphate [77] of the NTP were also included. The coordination between the Mg^2+^ and NTP was kept by adding the harmonic restraints with a force constant of 2261.03 kJ.mol^-1^.Å^-2^ between the magnesium ion and an oxygen atom attached to the P_γ_ atom. Since the binding of the Mg^2+^ to the NTP may induce significant charge re-distribution, we have regenerated the partial charges of the (NTP-Mg)^2-^ group using the restrained electrostatic potential (RESP) [78] fitting to the quantum calculation (HF/6-31G*). The partial charges of the (NTP-Mg)^2-^ group ((UTP-Mg)^2-^ for main channel and (ATP-Mg)^2-^ for secondary channel) are listed in S7 and S8 Tables. The quantum calculations were performed using Gaussion03 [79].

After building up the structural models, each system was then neutralized by adding 143 sodium ions and solvated in a water box containing 139,784 TIP3P water molecules [72]. Because in the previous channel analysis the (NTP-Mg)^2-^ group was simulated as a sphere without real geometrical shape, the (NTP-Mg)^2-^ group in the simulation models may clash with the surrounding residues. Furthermore, to avoid the inserted (NTP-Mg)^2-^ group from perturbing the Pol II conformation, we first froze the Pol II complex and only performed 1,000-steps energy minimization with the steepest descent algorithm on the (NTP-Mg)^2-^ group to let it re-orient and fit itself into the channels. To investigate the movement of the (NTP-Mg)^2-^ group, we measured the positional change for the c.o.m. of (NTP-Mg)^2-^ group for each energy minimization simulation (S2 Fig). We also calculated the shortest distance between the atoms of (NTP-Mg)^2-^ group and the initial reference center position (S2 Fig). After energy minimization of only the (NTP-Mg)^2-^ group in the channels, we performed 10,000-steps energy minimization on the whole system, followed by a 200ps position restrain simulation with a force constant of 10 kJ.mol^-1^.Å^-2^ on all the heavy atoms of the Pol II complex together with (NTP-Mg)^2-^ group under NVT ensemble (T = 310K). Afterwards, the restrain was released and one 10ns simulation was performed with random initial velocity under the NVT ensemble, and the simulated annealing algorithm was applied to elevate the temperature from 50K to 310K in the first 500ps of this simulation. Because we have 40 structural models in total and we performed one simulation for each model, finally we have 40 MD trajectories. Other simulation parameters are the same as those used for the MD simulations of Pol II elongation complex.

### Calculations of non-bonded interactions that NTP experiences

We collected the last 5ns MD conformations for the calculations. Because we saved the snapshots every 20ps, the ensemble contains 5,020 conformations for each channel. In the calculations, Pol II, (NTP-Mg)^2-^ and solvent were treated as three individual energy groups. For each MD conformation, we first calculated the Lennard-Jones (E__LJ_) and short-range Coulomb (E__Coul_SR_) interaction energies between the (NTP-Mg)^2-^ and Pol II. For the long-range Coulomb interaction, we first calculated the total PME energy of (NTP-Mg)^2-^ and Pol II (E_PME_NTP+PolII_) by turning off the partial charges of solvent. Afterwards, we set the partial charges of (NTP-Mg)^2-^ and solvent to zero to calculate the PME energy of Pol II (E_PME_PolII_). Similarly, we turned off the partial charges of Pol II and solvent to calculate the PME energy of (NTP-Mg)^2-^ (E_PME_NTP_). The long-range electrostatic interaction energy (E__Coul_LR_) between (NTP-Mg)^2-^ and Pol II was then derived by subtracting (E_PME_PolII_) and (E_PME_NTP_) from (E_PME_NTP+PolII_). By summing up E__LJ_, E__Coul_SR_ and E__Coul_LR_, we could get the non-bonded interaction energy between (NTP-Mg)^2-^ and Pol II. Similar procedure was applied to calculate the non-bonded interaction energy between (NTP-Mg)^2-^ and solvent. The non-bonded interaction energy between (NTP-Mg)^2-^ and environment was then obtained by summing up the interaction energy between (NTP-Mg)^2-^ and Pol II/solvent. To demonstrate the non-bonded interaction energy in both channels, we used bootstrapping algorithm to randomly select 100 conformations from the ensemble and repeated it 100 times to obtain the average values (Fig 6). To investigate the non-bonded interaction energies that NTP experiences along the pathways, we divided the conformations from the last 5ns MD simulations into 20 clusters according to the (NTP-Mg)^2-^ positional similarity in the channels. We then calculated the non-bonded interaction energy using the 20 MD cluster center conformations for each channel (S3 Fig).

### Calculations of charges around NTP

We calculated the net charge within certain radius of NTP for the Pol II complex (Fig 7). The MD conformations from the last 5ns simulations (5,020 conformations per channel) were used for the calculations. In particular, for one MD conformation, the charge of the Pol II complex was calculated by considering charged amino acids and nucleotides with any atom inside a certain radius of NTP atoms. We repeated the calculation by changing the distance cut-off from 2Å to 10Å. Afterwards, a bootstrapping algorithm was applied to randomly select 100 conformations from the MD conformational ensemble, and this process was repeated 100 times to get the averaged net charges of the Pol II complex (Fig 7A). To study more details about the charges of the Pol II complex, we applied the same calculation method for three separated groups: nucleotides, positive-charged amino acids (LYS, ARG and HIP) and negative-charged amino acids (GLU and ASP) (Figs 7B–7D). In addition, we also calculated the charge of sodium (Na^+^) by using the same method with the distance cut-off 10Å.

## Supporting Information

S1 Text Structural model of the Pol II elongation complex with the complete transcription bubble. (PDB)

S2 Text Supplementary information text. (DOC)

S1 Fig RMSD of the alpha carbon atoms in the four parallel 20ns MD simulations. (TIF)

S2 Fig Distance between the NTP and the caver centers after energy minimization. (a) Distance between NTP and caver center for the main channel. The red circles with solid line show the distance of NTP center of mass to the corresponding caver center, while the black triangles with dashed lines demonstrate the shortest distance of NTP to the caver center. (b) The same as (a) but for the secondary channel.
(TIF)

S3 Fig Non-bonded interactions between NTP and the environment revealed by MD conformations. (a) Coulomb interactions between NTP and environment along different positions of the main channel (red solid line with triangles) and secondary channel (black dashed line with circles). (b) Similar to (a) but for the Lennard-Jones energies.
(TIF)

S4 Fig Electrostatic potential along the NTP channels. (a) The surface of the protein and nucleotides within 10Å of the main channel NTP diffusion pathway are shown colored by its electrostatic potential. Amino acids at >10Å from the main channel are shown just in a surface representation and grey color. The template DNA, non-template DNA and RNA strand are shown in licorice representation (cyan, green and red colors, respectively). The right panel is a rotation of 180° respect to the left panel. The empty arrow denotes the NTP pathway. (b) The same as (a) but for the secondary channel.
(TIF)

S5 Fig Spt4/5 elongation factor binding to the upstream DNA locates near the entry of main channel pathway. In (a)-(c), the main channel pathway discovered is shown in yellow with different views (side view (a), front view (b) and top view (c)). The template DNA (cyan), non-template DNA (green) and RNA (red) strand are shown with tube and licorice representations. The Pol II protein components are shown in grey; (d)-(f) are similar to (a)-(c), but with the elongation factor Spt4 and Spt5 shown in pink and blue, respectively.
(TIF)

S6 Fig Distribution of counter ion Na^+^ in the vicinity of Nucleic Acids during MD simulation. (a) Left and right panels show the distribution of sodium ions (blue sphere) before and after the MD simulation. The two bottom boxes are close-ups of the nucleotides and ions. The template DNA (cyan), non-template DNA (green) and RNA (red) strand are shown in tube and licorice representations. Protein surface is shown in light grey. (b) The plot shows the number of Na^+^ ions within 9Å of the nucleotides during the course of 4 independent MD simulations.
(TIF)

S7 Fig Probability of consistent protonation states in our MD setup and the pKa predicted by Propka3.1. The values were obtained by considering 44 MD conformations. Please refer to S1–S5 Tables for the residue index.
(TIF)

S1 Table Comparison between protonation states of aspartic acid (ASP) adopted in our MD simulations and those predicted by the Propka software. <pKa> were obtained by averaging predictions made by the Propka software using 44 MD conformations.
(DOC)

S2 Table Comparison between protonation states of glutamic acid (GLU) adopted in our MD simulations and those predicted by the Propka software. <pKa> were obtained by averaging predictions made by the Propka software using 44 MD conformations. Amino acids whose predicted pKa value (Propka) suggests a protonation state that differs from the one used in our MD simulations are highlighted.
(DOC)

S3 Table Comparison between protonation states of lysine (LYS) adopted in our MD simulations and those predicted by the Propka software. <pKa> were obtained by averaging predictions made by the Propka software using 44 MD conformations. Amino acids whose predicted pKa value (Propka) suggests a protonation state that differs from the one used in our MD simulations are highlighted.
(DOC)

S4 Table Comparison between protonation states of arginine (ARG) adopted in our MD simulations and those predicted by the Propka software. <pKa> were obtained by averaging predictions made by the Propka software using 44 MD conformations.
(DOC)

S5 Table Comparison between protonation states of histidine (HIS) adopted in our MD simulations and those predicted by the Propka software. <pKa> were obtained by averaging predictions made by the Propka software using 44 MD conformations. Amino acids whose predicted pKa value (Propka) suggests a protonation state that differs from the one used in our MD simulations are highlighted.
(DOC)

S6 Table Distances between the amino acids and the main/secondary channel. The table presents the distance to the NTP diffusion pathways for the six amino acids whose predicted pKa value (Propka) suggests a protonation state that differs from the one used in our MD simulations. The distances are the average minimum distance from the 44 MD conformations used for Propka predictions.
(DOC)

S7 Table Partial charges of (UTP-Mg)^2-^ group. (DOC)

S8 Table Partial charges of (ATP-Mg)^2-^ group. (DOC)
