# Supplementary material for: Structural Model of RNA Polymerase II Elongation Complex with Complete Transcription Bubble Reveals NTP Entry Routes
Source: PLoS Comput Biol. 2015 Jul 2;11(7):e1004354. doi: 10.1371/journal.pcbi.1004354 (PMC4489626; doi:10.1371/journal.pcbi.1004354)
Supplement: S2 Text — (DOC) [file pcbi.1004354.s002.doc]

***Supporting Information***

**Position-dependent Energy Analysis**

In order to investigate the position-dependent energies under dynamic protein environment, we collected the conformations from the last 5ns of the 20×10ns simulations (5020 snapshots for each channel). For each MD conformation, we calculated the minimum distance between the NTP and its pairing partner in the binding site (dA at i+2 site and dT at i+1 site in the template strand for the main channel and secondary channel, respectively) and non-bonded energy experienced by NTP. Afterwards, we divided the conformations into 3 groups according to the distances from the NTP to the binding site. For the conformations in each group, we calculated the average Coulomb and Lennard-Jones energies (S3 Fig.).

In the main channel, there is an increment of ~50kJ/mol for the Coulomb energy from the protein surface to the binding site (S3a Fig.). In contrast, there is a significant energy decrease (~130kJ/mol) along the secondary channel, especially around the active site (S3a Fig.). This difference may be due to the drastically different electrostatic environment near the end of the main channel (a large number of negatively charged nucleotides near the i+2 site, see S4a Fig.) and that of the secondary channel (a distribution of both positively and negatively charged protein residues near the active site, see S4b Fig.). This discrepancy between the two channels clearly suggests that the secondary channel is the more favorable route for NTP diffusion. Calculations of the Lennard-Jones energies along the two channels show a tendency of energy decrease from the protein surface to the binding site (S3b Fig.).

**Electrostatic Potential along the Channels**

The electrostatic potential for the Pol II complex was calculated using the APBS program [1] (S4 Fig.). The solute and solvent dielectric constants were set to 2.0 and 78.0, respectively. A temperature of 310K was used in the calculation.

**Channel Analysis with Elongation Factor Spt4/5**

***Model building***

Starting from an available all-atom model of the eukaryotic RNAP II elongation complex containing a full transcription bubble with Spt4/5 bound to the clamp domain [2] (quoted as “Pol II-Spt4/5” in the main text) we first made an structural alignment based on the protein backbones of residues 200~350 in chain A between our model (S1 Text.pdb) and the Pol II-Spt4/5 model. Afterwards, the elongation factors Spt4 (chain X) and Spt5 (chain Y) were extracted from the aligned PolII-Spt4/5 model and inserted into our Pol II energy-minimized model (S1 Text.pdb). The missing side-chains of amino acids in Spt4 and Spt5 were rebuilt using the WHAT IF online server (http://swift.cmbi.ru.nl/servers/html/index.html). The whole system was then solvated with TIP3P water molecules [3] in a box of 160Å*188Å*160Å (α=90°, β=90°, γ=90°). Enough sodium ions were added to neutralize the system. Finally, the simulation system contained 486,637 atoms.

***Molecular dynamics simulation***

To maintain the coordination between the zinc and the protein, harmonic restraints with a force constant of 2261.03 kJ.mol^-1^.Å^-2^ were added between the zinc ion and its coordinated cysteine residues (chain X and residues 7, 10, 24 and 27). Energy minimization was performed for the whole system (10,000 steps). Subsequently, we performed a position-restrained MD simulation to allow relaxation of the system, briefly: a restrain with a force constant of 10 kJ.mol^-1^.Å^-2^ was enforced on all the heavy atoms of the Pol II complex and the elongation factor Spt4/5, and the whole system was simulated for 200ps under an NVT ensemble (T=310K), followed by another 500ps simulation under an NPT ensemble (T=310K and P=1bar). Afterwards, the restrain was released, and molecular dynamics (MD) simulations were performed at 310K under the NVT ensemble. Three independent simulations with different initial velocities and a total integration time of 20ns were performed starting from the last configuration of the position restraint simulation. The first 500ps of simulation were used to gradually increase the temperature of the system from 50K to 310K. We recorded snapshots of the complete system every 20ps. Other parameters are the same as those in the “MD simulations of Pol II elongation complex” of the “Methods” section.

***Channel analysis***

To investigate the effect of elongation factor Spt4/5 on the NTP loading mechanism under the dynamic protein environment, the last 5ns of MD simulations with Spt4/5 (753 MD snapshots) were used to search for cavities that might form pathways connecting the protein surface to the i+2 binding site. The input parameters for CAVER [4] and the methodology for probability calculation are the same as those described in the subsection “Channel analysis based on MD conformations” of the “Methods” section.

Similar to our previous analysis without the Spt4/5 factor, we still found NTP may diffuse to the i+2 binding site via the pathways at both sides of the non-template DNA strand in the transcription bubble (S5 Fig.). Nevertheless, a difference is that the two pathways do not re-unit as one pathway at the protein surface (S5d-f Figs.). This could be explained since the location of the pathways union is taken by the elongation factor Spt4/5. Furthermore, when Spt4/5 is bound to the Pol II we also found that the probability of finding the main channel pathway is reduced from 31% to 24%.

In summary, although there is still enough space for NTP diffusion through the main channel pathway in the presence of elongation factor Spt4/5, its probability is reduced and instead of one bifurcated pathway, there are two separated channels reach the protein surface.

**Distribution of Counter Ions**

When preparing the system, we randomly placed these counter ions in the simulation box. Ions were allowed to freely diffuse during MD simulations. Although we did not manually put more Na^+^ ions near the DNA/RNA region, after equilibration it can be observed that Na+ ions condensate in the proximity to the negatively charged nucleotide backbones (S6 Fig.). As shown in the S6a Fig., the counter ions are evenly distributed before the MD simulation. During the course of MD simulations, the counter ions accumulate around the nucleotides, likely due to the electrostatic attraction from the highly negative-charged nucleotides’ backbones. Furthermore, we found the number of Na^+^ ions reaches a plateau after 5ns, suggesting that Na^+^ distribution has reached the equilibrium at that point (S6b Fig.). Hence, we believe that our simulations are sufficient to allow a proper distribution of the ions in the simulation box.

**Protonation States of Titratable Residues**

Regarding the protonation states of tritatable residues, we adopted the same protonation states as in our previous work [5]. Due to the presence of large number of DNA/RNA motifs, the titratable residues may experience pKa shifts and thus take non-standard protonation states. Furthermore, the protonation states may even vary under dynamic protein environment. Therefore, in order to investigate the validity of the protonation states used for titratable residues, we adopted the program Propka3.1 [6, 7] to calculate the pKa values and predict the protonation states for Asp, Glu, Lys, Arg and His using 44 representative conformations (11 conformations separated by 500ps from the last 5ns of the 4×20ns MD simulations) (S3-S7 Tables). The predicted protonation states of >1000 titratable residues from the averaged pKa values agree well with what we used in our MD simulations, except for four residues: glutamic acid E1426 (chain A), lysine K979 (chain B) and two histidines H1085 (chain A) and H91 (chain C) (highlighted in S2, S3 and S5 Tables). In order to investigate the effect of a dynamic protein conformational ensemble on the protonation states, we have also studied the probability of the consistent protonation states adopted in our MD simulations and those predicted by the program Propka3.1 (S7 Fig.). The results indicate that the predicted protonation states for all the Asp and Arg residues match with what we used in our simulations (S7 Fig.). For Glu, Lys and His, their protonation states display small variations, but in general agree well with those used in our MD simulations (over 60%) (S7 Fig.). The exceptions are: E1426 (chain A), H1085 (chain A), H91 (chain C), H734 (chain B), H146 (chain E) and K979 (chain B) (highlighted in S2, S3 and S5 Tables). However, we found that all these 6 residues with inconsistent protonation states are all far away from any of the two NTP diffusion channels (see S6 Table), which suggest that their protonation states may not significantly affect the described NTP loading mechanism.

**References**

1. Baker NA, Sept D, Joseph S, Holst MJ, McCammon JA. Electrostatics of nanosystems: application to microtubules and the ribosome. Proc Natl Acad Sci U S A. 2001;98(18):10037-41. doi: 10.1073/pnas.181342398. PubMed PMID: 11517324; PubMed Central PMCID: PMC56910.

2. Martinez-Rucobo FW, Sainsbury S, Cheung ACM, Cramer P. Architecture of the RNA polymerase-Spt4/5 complex and basis of universal transcription processivity. Embo J. 2011;30(7):1302-10. doi: Doi 10.1038/Emboj.2011.64. PubMed PMID: WOS:000290305200014.

3. Jorgensen WL, Chandrasekhar J, Madura JD, Impey RW, Klein ML. Comparison of Simple Potential Functions for Simulating Liquid Water. J Chem Phys. 1983;79(2):926-35. PubMed PMID: ISI:A1983QZ31500046.

4. Chovancova E, Pavelka A, Benes P, Strnad O, Brezovsky J, Kozlikova B, et al. CAVER 3.0: A Tool for the Analysis of Transport Pathways in Dynamic Protein Structures. Plos Computational Biology. 2012;8(10). doi: Artn E1002708

Doi 10.1371/Journal.Pcbi.1002708. PubMed PMID: WOS:000310568800009.

5. Silva DA, Weiss DR, Pardo-Avila F, Da LT, Levitt M, Wang D, et al. Millisecond dynamics of RNA polymerase II translocation at atomic resolution. Proceedings of the National Academy of Sciences of the United States of America. 2014;111(21):7665-70. doi: Doi 10.1073/Pnas.1315751111. PubMed PMID: WOS:000336411300044.

6. Olsson MHM, Sondergaard CR, Rostkowski M, Jensen JH. PROPKA3: Consistent Treatment of Internal and Surface Residues in Empirical pK(a) Predictions. Journal of Chemical Theory and Computation. 2011;7(2):525-37. doi: Doi 10.1021/Ct100578z. PubMed PMID: WOS:000287049200025.

7. Sondergaard CR, Olsson MHM, Rostkowski M, Jensen JH. Improved Treatment of Ligands and Coupling Effects in Empirical Calculation and Rationalization of pK(a) Values. Journal of Chemical Theory and Computation. 2011;7(7):2284-95. doi: Doi 10.1021/Ct200133y. PubMed PMID: WOS:000292617900029.
