# Supplementary figures and images for: Structural Model of RNA Polymerase II Elongation Complex with Complete Transcription Bubble Reveals NTP Entry Routes
Source: PLoS Comput Biol. 2015 Jul 2;11(7):e1004354. doi: 10.1371/journal.pcbi.1004354 (PMC4489626; doi:10.1371/journal.pcbi.1004354)

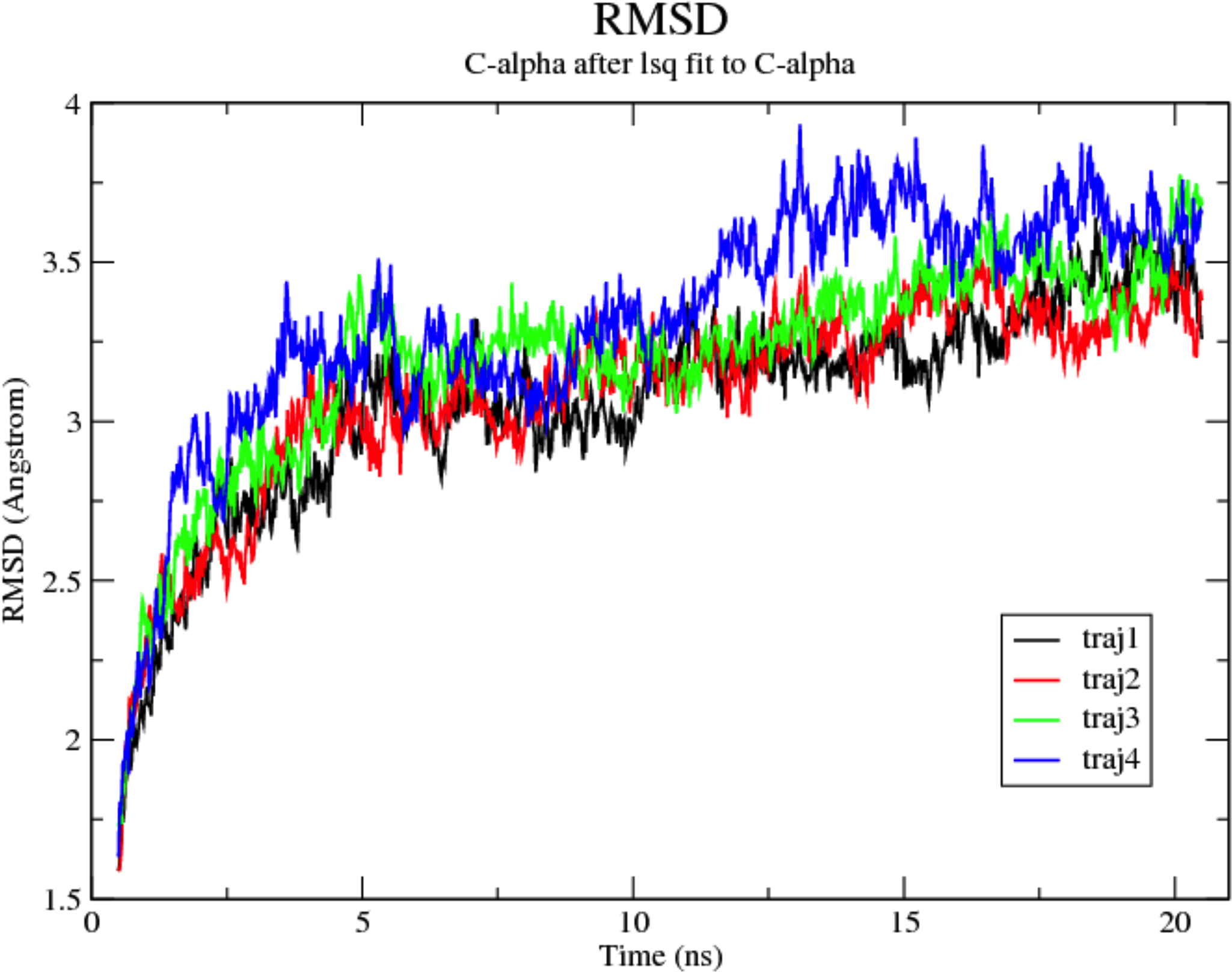

Supplement: S1 Fig — (TIF) [file pcbi.1004354.s003.tif]

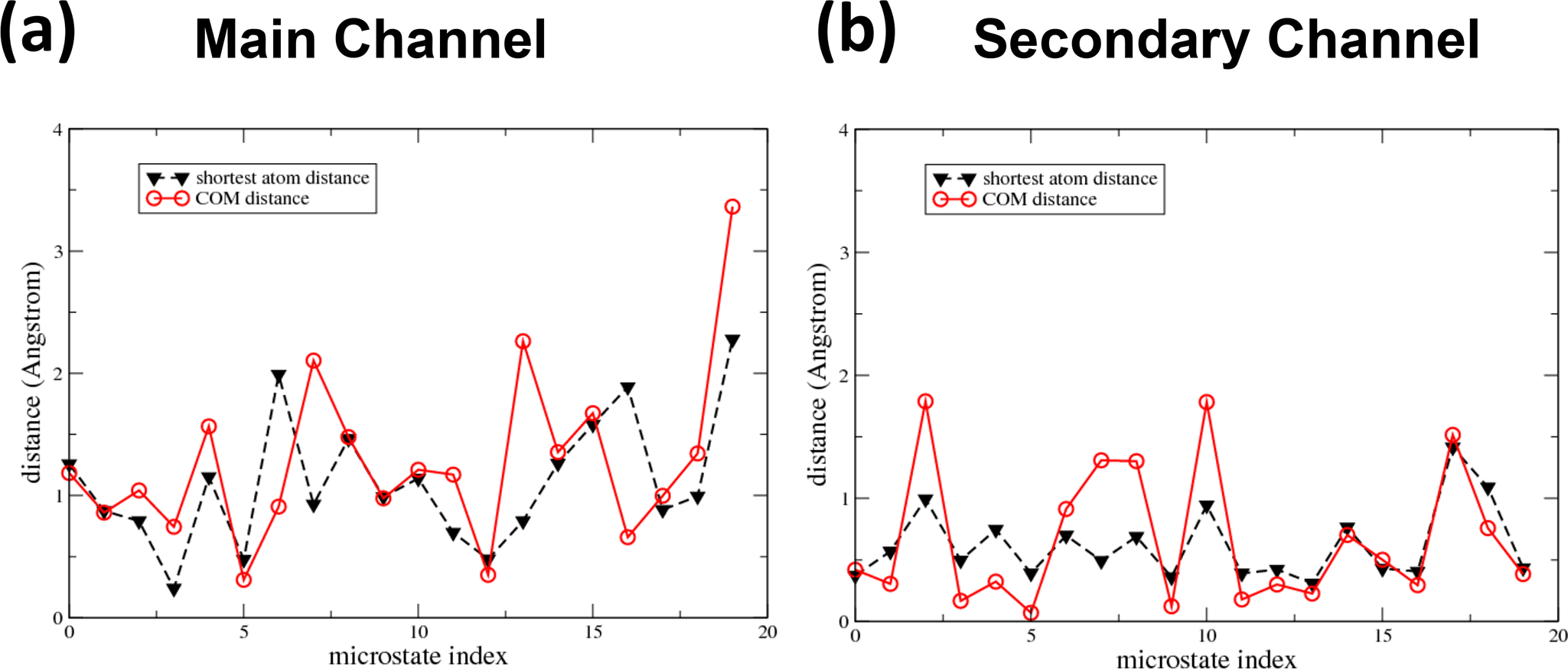

Supplement: S2 Fig — (a) Distance between NTP and caver center for the main channel. The red circles with solid line show the distance of NTP center of mass to the corresponding caver center, while the black triangles with dashed lines demonstrate the shortest distance of NTP to the caver center. (b) The same as (a) but for the secondary channel. (TIF) [file pcbi.1004354.s004.tif]

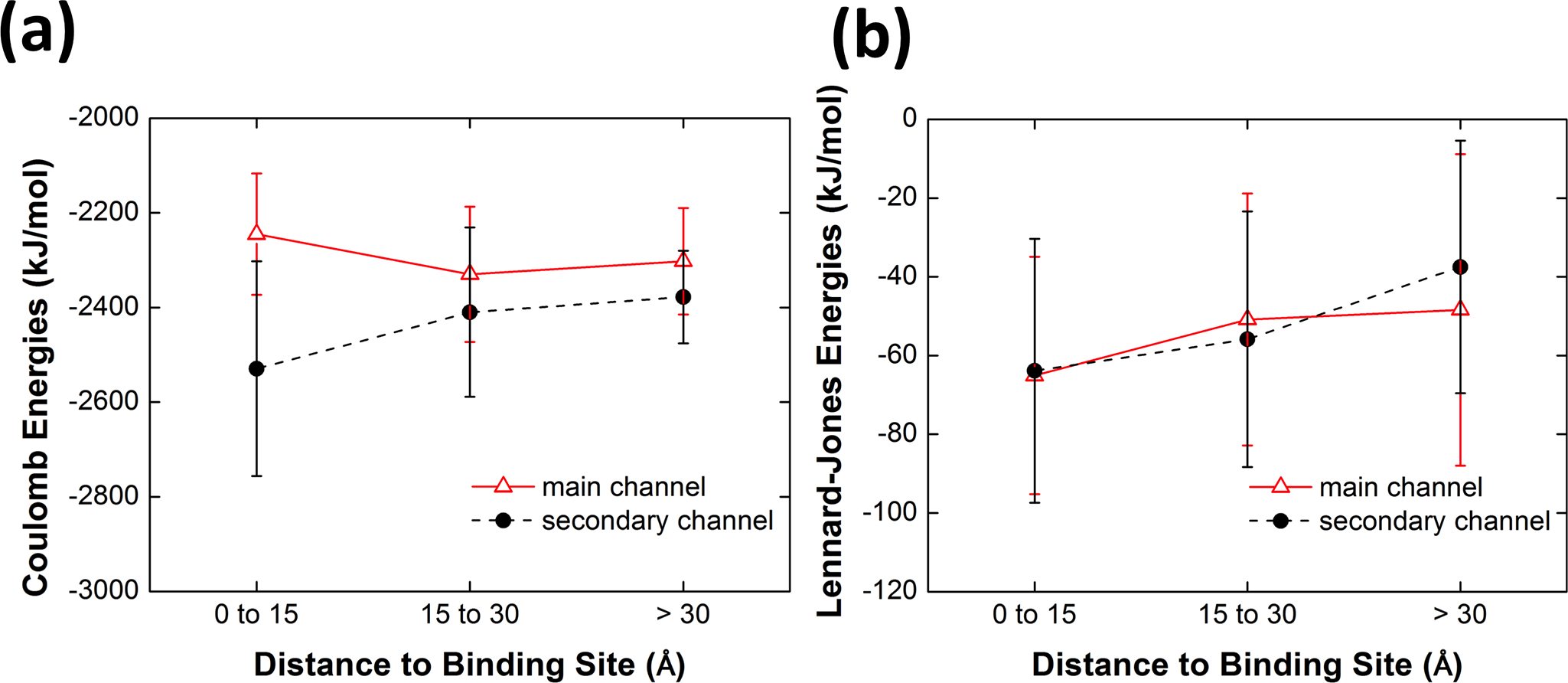

Supplement: S3 Fig — (a) Coulomb interactions between NTP and environment along different positions of the main channel (red solid line with triangles) and secondary channel (black dashed line with circles). (b) Similar to (a) but for the Lennard-Jones energies. (TIF) [file pcbi.1004354.s005.tif]

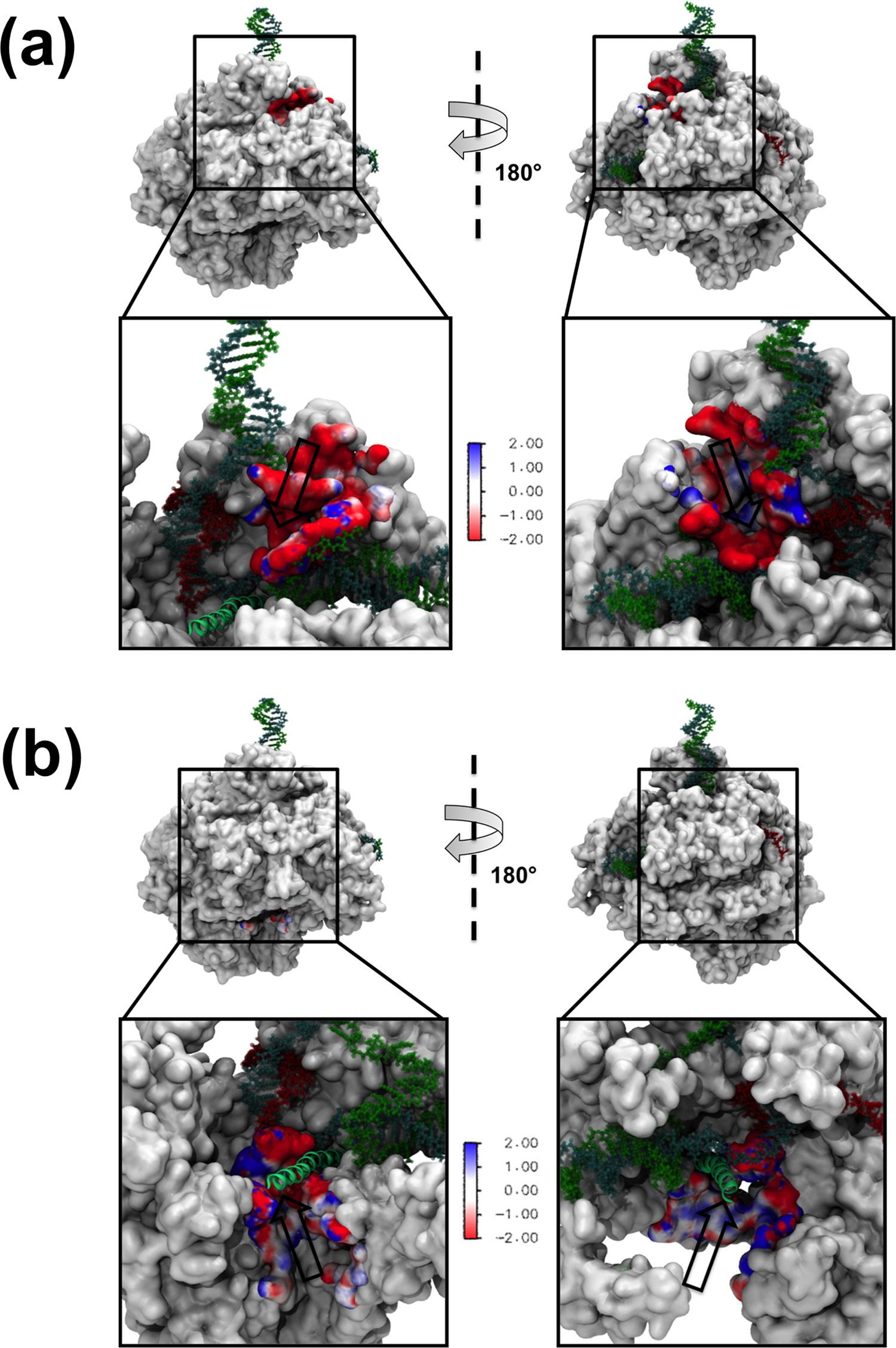

Supplement: S4 Fig — (a) The surface of the protein and nucleotides within 10Å of the main channel NTP diffusion pathway are shown colored by its electrostatic potential. Amino acids at >10Å from the main channel are shown just in a surface representation and grey color. The template DNA, non-template DNA and RNA strand are shown in licorice representation (cyan, green and red colors, respectively). The right panel is a rotation of 180° respect to the left panel. The empty arrow denotes the NTP pathway. (b) The same as (a) but for the secondary channel. (TIF) [file pcbi.1004354.s006.tif]

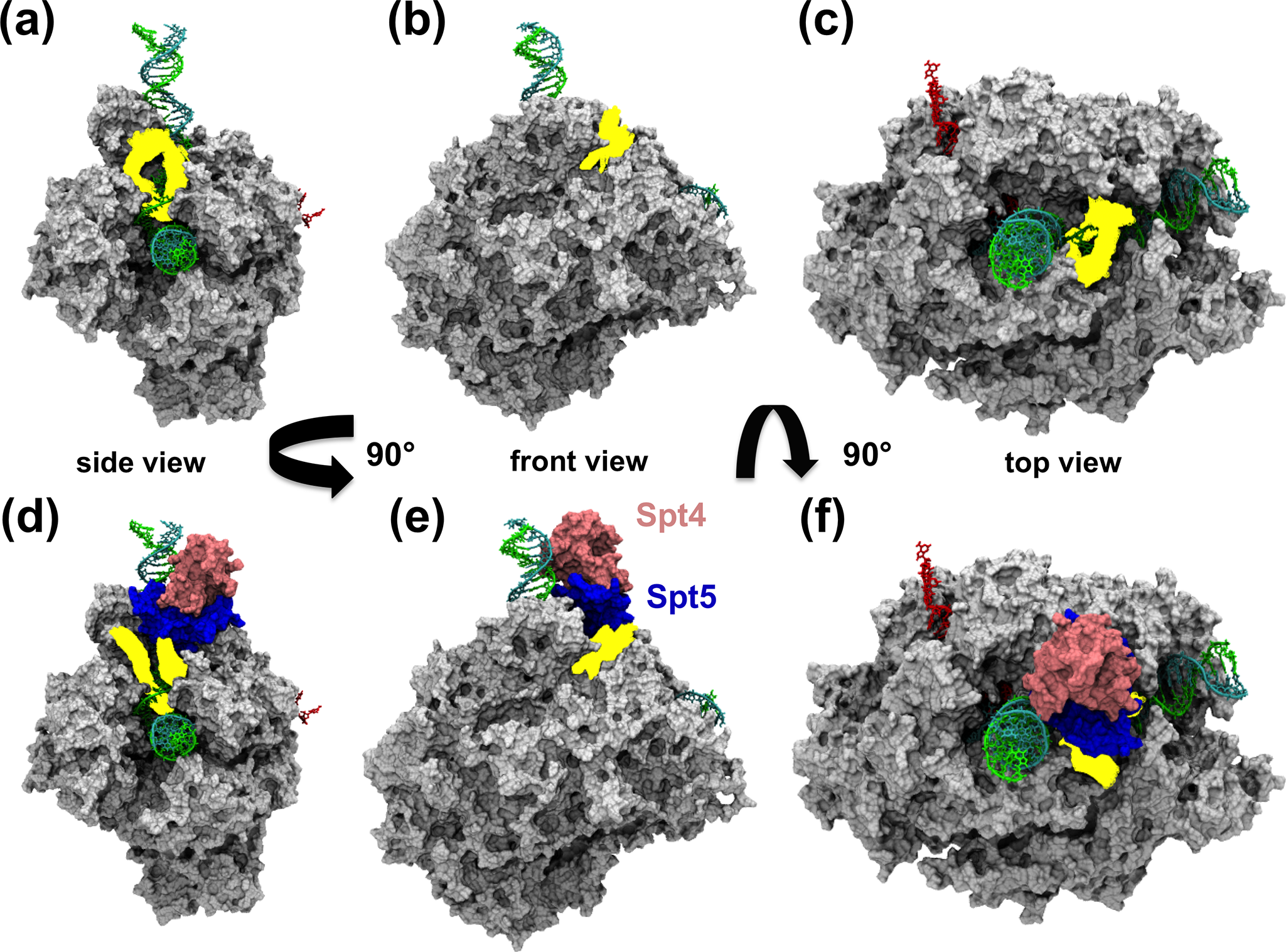

Supplement: S5 Fig — In (a)-(c), the main channel pathway discovered is shown in yellow with different views (side view (a), front view (b) and top view (c)). The template DNA (cyan), non-template DNA (green) and RNA (red) strand are shown with tube and licorice representations. The Pol II protein components are shown in grey; (d)-(f) are similar to (a)-(c), but with the elongation factor Spt4 and Spt5 shown in pink and blue, respectively. (TIF) [file pcbi.1004354.s007.tif]

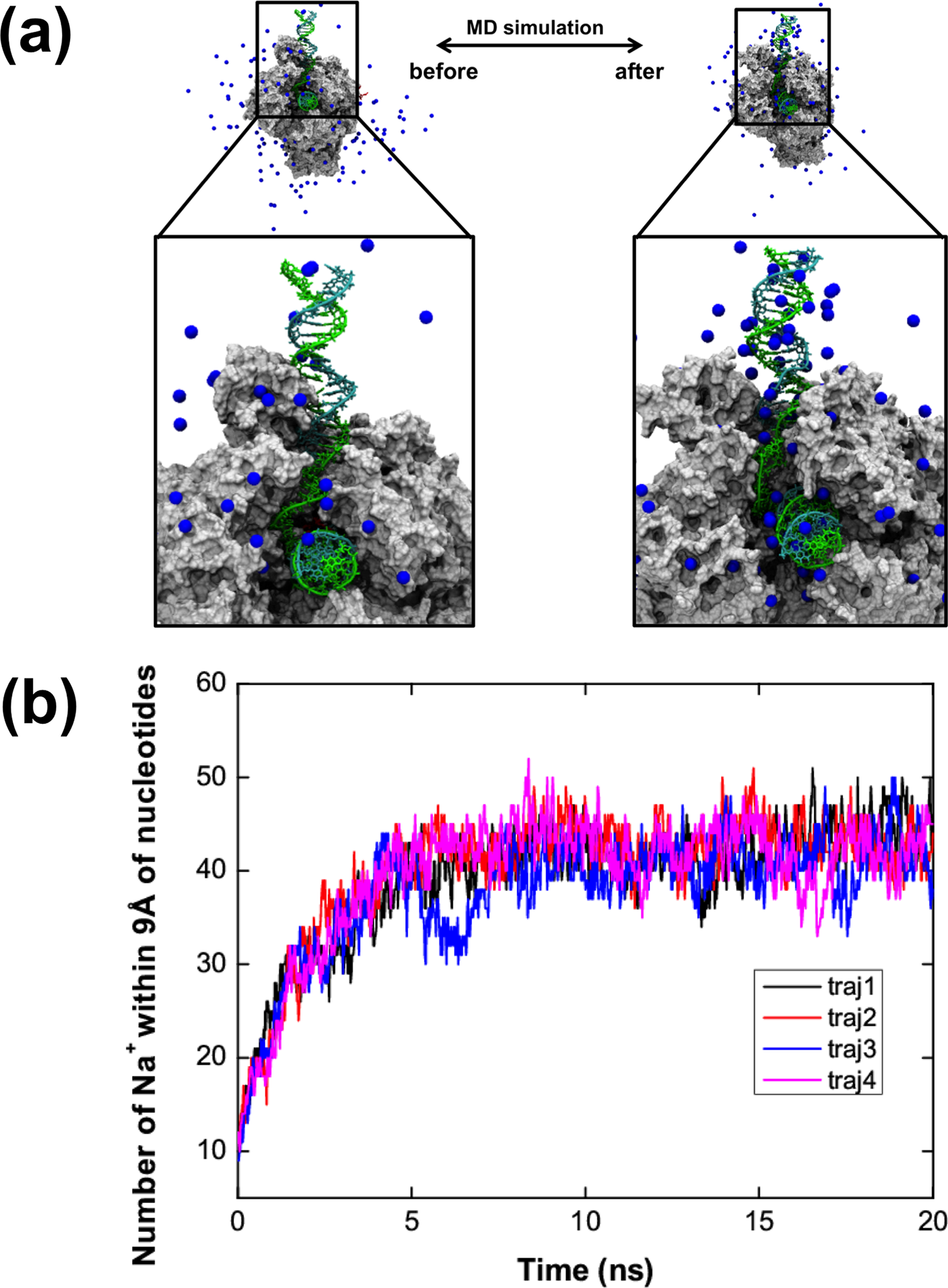

Supplement: S6 Fig — (a) Left and right panels show the distribution of sodium ions (blue sphere) before and after the MD simulation. The two bottom boxes are close-ups of the nucleotides and ions. The template DNA (cyan), non-template DNA (green) and RNA (red) strand are shown in tube and licorice representations. Protein surface is shown in light grey. (b) The plot shows the number of Na+ ions within 9Å of the nucleotides during the course of 4 independent MD simulations. (TIF) [file pcbi.1004354.s008.tif]

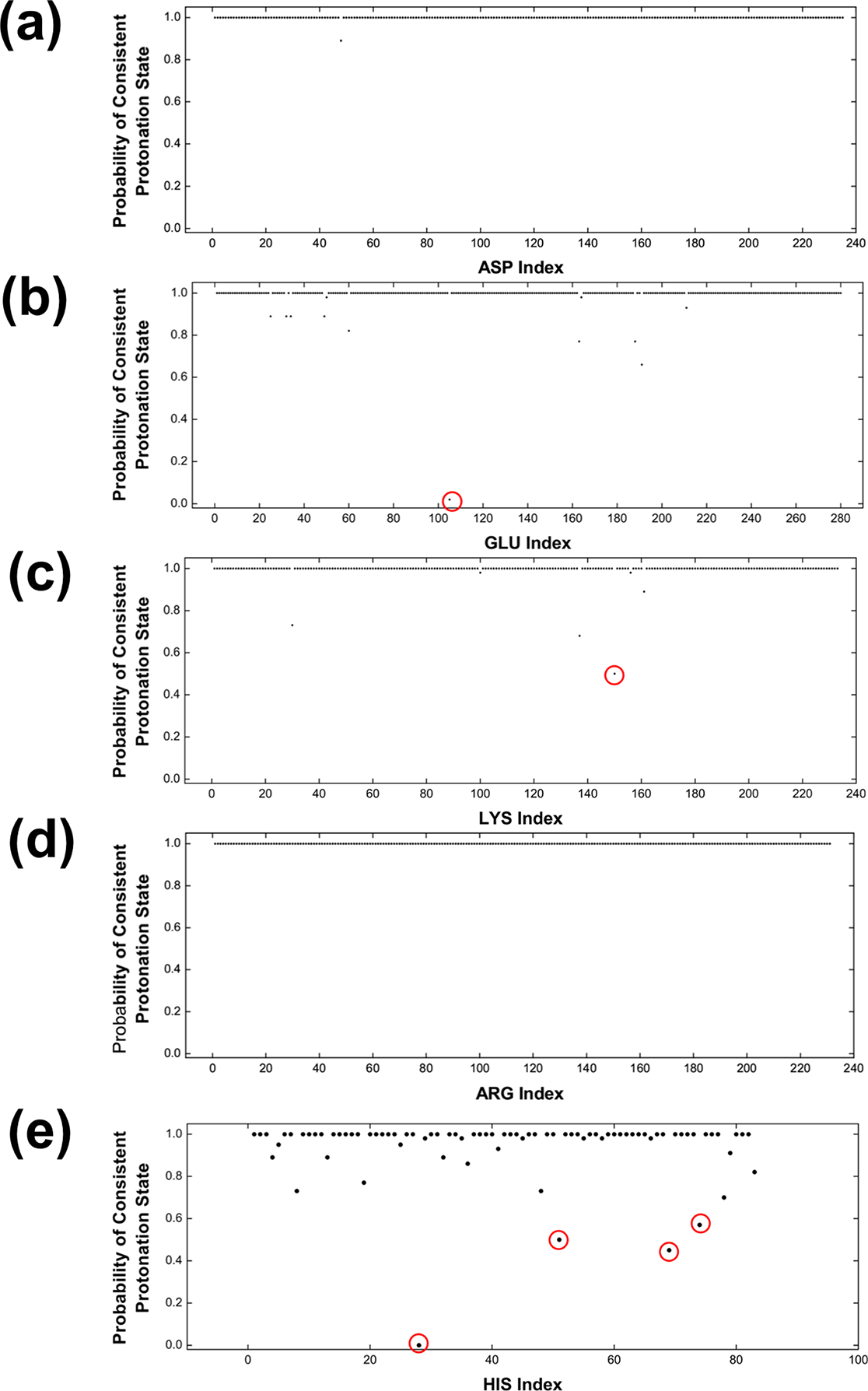

Supplement: S7 Fig — The values were obtained by considering 44 MD conformations. Please refer to S1–S5 Tables for the residue index. (TIF) [file pcbi.1004354.s009.tif]
