# Supplementary material for: Structural Model of RNA Polymerase II Elongation Complex with Complete Transcription Bubble Reveals NTP Entry Routes
Source: PLoS Comput Biol. 2015 Jul 2;11(7):e1004354. doi: 10.1371/journal.pcbi.1004354 (PMC4489626; doi:10.1371/journal.pcbi.1004354)
Supplement: S1 Table — were obtained by averaging predictions made by the Propka software using 44 MD conformations. (DOC) [file pcbi.1004354.s010.doc]

**S1** **Table** **Comparison between protonation states of aspartic acid (ASP) adopted in our MD simulations and those predicted by the Propka software.** <pKa> were obtained by averaging predictions made by the Propka software using 44 MD conformations.

| ASP Index | residue ID | chain ID | <pKa> | predicted state | used state | ASP Index | residue ID | chain ID | <pKa> | predicted state | used state |
| --- | --- | --- | --- | --- | --- | --- | --- | --- | --- | --- | --- |
| 1 | 42 | A | 4.17 | ASP | ASP | 50 | 826 | A | 4.10 | ASP | ASP |
| 2 | 55 | A | 4.08 | ASP | ASP | 51 | 847 | A | 4.87 | ASP | ASP |
| 3 | 62 | A | 3.51 | ASP | ASP | 52 | 853 | A | 1.99 | ASP | ASP |
| 4 | 85 | A | 3.31 | ASP | ASP | 53 | 871 | A | 4.26 | ASP | ASP |
| 5 | 116 | A | 3.40 | ASP | ASP | 54 | 874 | A | 3.50 | ASP | ASP |
| 6 | 130 | A | 2.96 | ASP | ASP | 55 | 884 | A | 1.67 | ASP | ASP |
| 7 | 151 | A | 3.37 | ASP | ASP | 56 | 890 | A | 3.31 | ASP | ASP |
| 8 | 156 | A | 3.36 | ASP | ASP | 57 | 900 | A | 2.49 | ASP | ASP |
| 9 | 157 | A | 3.74 | ASP | ASP | 58 | 905 | A | 2.82 | ASP | ASP |
| 10 | 177 | A | 3.30 | ASP | ASP | 59 | 909 | A | 2.52 | ASP | ASP |
| 11 | 188 | A | 3.10 | ASP | ASP | 60 | 922 | A | 3.11 | ASP | ASP |
| 12 | 193 | A | 3.57 | ASP | ASP | 61 | 930 | A | 3.19 | ASP | ASP |
| 13 | 195 | A | 2.98 | ASP | ASP | 62 | 939 | A | 4.53 | ASP | ASP |
| 14 | 218 | A | 3.78 | ASP | ASP | 63 | 949 | A | 3.33 | ASP | ASP |
| 15 | 260 | A | 2.36 | ASP | ASP | 64 | 974 | A | 1.79 | ASP | ASP |
| 16 | 261 | A | 3.83 | ASP | ASP | 65 | 980 | A | 3.23 | ASP | ASP |
| 17 | 268 | A | 3.98 | ASP | ASP | 66 | 985 | A | 3.70 | ASP | ASP |
| 18 | 305 | A | 3.49 | ASP | ASP | 67 | 992 | A | 4.06 | ASP | ASP |
| 19 | 307 | A | 4.35 | ASP | ASP | 68 | 1013 | A | 4.06 | ASP | ASP |
| 20 | 346 | A | 3.39 | ASP | ASP | 69 | 1043 | A | 4.00 | ASP | ASP |
| 21 | 356 | A | 3.26 | ASP | ASP | 70 | 1127 | A | 3.37 | ASP | ASP |
| 22 | 362 | A | 3.98 | ASP | ASP | 71 | 1155 | A | 4.77 | ASP | ASP |
| 23 | 386 | A | 3.91 | ASP | ASP | 72 | 1157 | A | 2.39 | ASP | ASP |
| 24 | 408 | A | 3.71 | ASP | ASP | 73 | 1166 | A | 3.72 | ASP | ASP |
| 25 | 411 | A | 2.95 | ASP | ASP | 74 | 1178 | A | 3.35 | ASP | ASP |
| 26 | 414 | A | 3.08 | ASP | ASP | 75 | 1186 | A | 3.63 | ASP | ASP |
| 27 | 423 | A | 3.42 | ASP | ASP | 76 | 1198 | A | 3.86 | ASP | ASP |
| 28 | 438 | A | 3.66 | ASP | ASP | 77 | 1204 | A | 1.55 | ASP | ASP |
| 29 | 440 | A | 3.94 | ASP | ASP | 78 | 1206 | A | 3.50 | ASP | ASP |
| 30 | 481 | A | 3.75 | ASP | ASP | 79 | 1223 | A | 4.05 | ASP | ASP |
| 31 | 483 | A | 3.99 | ASP | ASP | 80 | 1231 | A | 3.26 | ASP | ASP |
| 32 | 485 | A | 0.10 | ASP | ASP | 81 | 1233 | A | 3.18 | ASP | ASP |
| 33 | 526 | A | 5.15 | ASP | ASP | 82 | 1249 | A | 2.62 | ASP | ASP |
| 34 | 538 | A | 4.20 | ASP | ASP | 83 | 1257 | A | 4.86 | ASP | ASP |
| 35 | 544 | A | 1.45 | ASP | ASP | 84 | 1288 | A | 3.42 | ASP | ASP |
| 36 | 555 | A | 3.44 | ASP | ASP | 85 | 1309 | A | 3.53 | ASP | ASP |
| 37 | 557 | A | 3.45 | ASP | ASP | 86 | 1323 | A | 3.48 | ASP | ASP |
| 38 | 592 | A | 3.13 | ASP | ASP | 87 | 1334 | A | 3.89 | ASP | ASP |
| 39 | 602 | A | 3.32 | ASP | ASP | 88 | 1359 | A | 3.45 | ASP | ASP |
| 40 | 609 | A | 3.55 | ASP | ASP | 89 | 1373 | A | 2.88 | ASP | ASP |
| 41 | 668 | A | 5.50 | ASP | ASP | 90 | 1419 | A | 5.09 | ASP | ASP |
| 42 | 672 | A | 2.86 | ASP | ASP | 91 | 1420 | A | 3.01 | ASP | ASP |
| 43 | 692 | A | 3.71 | ASP | ASP | 92 | 1442 | A | 3.73 | ASP | ASP |
| 44 | 716 | A | 2.82 | ASP | ASP | 93 | 20 | B | 2.78 | ASP | ASP |
| 45 | 727 | A | 3.38 | ASP | ASP | 94 | 29 | B | 2.93 | ASP | ASP |
| 46 | 739 | A | 3.63 | ASP | ASP | 95 | 49 | B | 4.27 | ASP | ASP |
| 47 | 781 | A | 2.63 | ASP | ASP | 96 | 56 | B | 3.71 | ASP | ASP |
| 48 | 790 | A | 5.99 | ASP | ASP | 97 | 61 | B | 4.25 | ASP | ASP |
| 49 | 791 | A | 3.73 | ASP | ASP | 98 | 66 | B | 4.37 | ASP | ASP |

| ASP Index | residue ID | chain ID | <pKa> | predicted state | used state | ASP Index | residue ID | chain ID | <pKa> | predicted state | used state |
| --- | --- | --- | --- | --- | --- | --- | --- | --- | --- | --- | --- |
| 99 | 82 | B | 2.81 | ASP | ASP | 148 | 909 | B | 2.94 | ASP | ASP |
| 100 | 106 | B | 3.50 | ASP | ASP | 149 | 921 | B | 3.20 | ASP | ASP |
| 101 | 131 | B | 3.84 | ASP | ASP | 150 | 936 | B | 3.14 | ASP | ASP |
| 102 | 141 | B | 3.35 | ASP | ASP | 151 | 950 | B | 2.04 | ASP | ASP |
| 103 | 158 | B | 2.71 | ASP | ASP | 152 | 959 | B | 3.79 | ASP | ASP |
| 104 | 159 | B | 3.48 | ASP | ASP | 153 | 978 | B | 5.23 | ASP | ASP |
| 105 | 188 | B | 3.07 | ASP | ASP | 154 | 998 | B | 0.00 | ASP | ASP |
| 106 | 198 | B | 2.81 | ASP | ASP | 155 | 1009 | B | 4.52 | ASP | ASP |
| 107 | 279 | B | 3.01 | ASP | ASP | 156 | 1043 | B | 4.07 | ASP | ASP |
| 108 | 294 | B | 4.38 | ASP | ASP | 157 | 1049 | B | 3.21 | ASP | ASP |
| 109 | 304 | B | 3.57 | ASP | ASP | 158 | 1100 | B | 3.93 | ASP | ASP |
| 110 | 307 | B | 2.89 | ASP | ASP | 159 | 1101 | B | 3.35 | ASP | ASP |
| 111 | 320 | B | 3.83 | ASP | ASP | 160 | 1125 | B | 5.19 | ASP | ASP |
| 112 | 326 | B | 2.62 | ASP | ASP | 161 | 1136 | B | 5.08 | ASP | ASP |
| 113 | 332 | B | 3.10 | ASP | ASP | 162 | 1156 | B | 3.98 | ASP | ASP |
| 114 | 354 | B | 3.63 | ASP | ASP | 163 | 1186 | B | 3.42 | ASP | ASP |
| 115 | 391 | B | 4.88 | ASP | ASP | 164 | 1190 | B | 3.48 | ASP | ASP |
| 116 | 394 | B | 4.28 | ASP | ASP | 165 | 1219 | B | 2.62 | ASP | ASP |
| 117 | 396 | B | 3.61 | ASP | ASP | 166 | 1223 | B | 3.87 | ASP | ASP |
| 118 | 397 | B | 1.29 | ASP | ASP | 167 | 16 | C | 3.39 | ASP | ASP |
| 119 | 399 | B | 4.03 | ASP | ASP | 168 | 19 | C | 4.10 | ASP | ASP |
| 120 | 407 | B | 4.57 | ASP | ASP | 169 | 26 | C | 4.69 | ASP | ASP |
| 121 | 427 | B | 4.33 | ASP | ASP | 170 | 47 | C | 1.36 | ASP | ASP |
| 122 | 441 | B | 3.40 | ASP | ASP | 171 | 60 | C | 2.27 | ASP | ASP |
| 123 | 505 | B | 3.22 | ASP | ASP | 172 | 76 | C | 3.08 | ASP | ASP |
| 124 | 550 | B | 3.39 | ASP | ASP | 173 | 85 | C | 3.94 | ASP | ASP |
| 125 | 568 | B | 4.07 | ASP | ASP | 174 | 90 | C | 2.67 | ASP | ASP |
| 126 | 576 | B | 3.32 | ASP | ASP | 175 | 93 | C | 3.38 | ASP | ASP |
| 127 | 608 | B | 3.90 | ASP | ASP | 176 | 117 | C | 3.59 | ASP | ASP |
| 128 | 618 | B | 3.74 | ASP | ASP | 177 | 136 | C | 2.66 | ASP | ASP |
| 129 | 629 | B | 4.07 | ASP | ASP | 178 | 181 | C | 3.91 | ASP | ASP |
| 130 | 642 | B | 3.61 | ASP | ASP | 179 | 190 | C | 4.65 | ASP | ASP |
| 131 | 643 | B | 3.85 | ASP | ASP | 180 | 196 | C | 3.23 | ASP | ASP |
| 132 | 668 | B | 3.97 | ASP | ASP | 181 | 211 | C | 3.93 | ASP | ASP |
| 133 | 675 | B | 3.87 | ASP | ASP | 182 | 217 | C | 3.70 | ASP | ASP |
| 134 | 694 | B | 4.25 | ASP | ASP | 183 | 220 | C | 2.64 | ASP | ASP |
| 135 | 709 | B | 4.27 | ASP | ASP | 184 | 226 | C | 3.77 | ASP | ASP |
| 136 | 720 | B | 4.00 | ASP | ASP | 185 | 241 | C | 4.19 | ASP | ASP |
| 137 | 722 | B | 3.31 | ASP | ASP | 186 | 249 | C | 2.95 | ASP | ASP |
| 138 | 724 | B | 3.48 | ASP | ASP | 187 | 266 | C | 2.92 | ASP | ASP |
| 139 | 760 | B | 3.79 | ASP | ASP | 188 | 268 | C | 3.89 | ASP | ASP |
| 140 | 790 | B | 3.90 | ASP | ASP | 189 | 2 | E | 2.74 | ASP | ASP |
| 141 | 837 | B | 3.65 | ASP | ASP | 190 | 25 | E | 4.37 | ASP | ASP |
| 142 | 847 | B | 1.42 | ASP | ASP | 191 | 41 | E | 3.18 | ASP | ASP |
| 143 | 861 | B | 5.16 | ASP | ASP | 192 | 48 | E | 2.38 | ASP | ASP |
| 144 | 891 | B | 3.63 | ASP | ASP | 193 | 74 | E | 3.43 | ASP | ASP |
| 145 | 894 | B | 2.48 | ASP | ASP | 194 | 84 | E | 3.57 | ASP | ASP |
| 146 | 895 | B | 2.60 | ASP | ASP | 195 | 159 | E | 3.16 | ASP | ASP |
| 147 | 896 | B | 3.68 | ASP | ASP | 196 | 182 | E | 5.89 | ASP | ASP |

| ASP Index | residue ID | chain ID | <pKa> | predicted state | used state | ASP Index | residue ID | chain ID | <pKa> | predicted state | used state |
| --- | --- | --- | --- | --- | --- | --- | --- | --- | --- | --- | --- |
| 197 | 77 | F | 3.22 | ASP | ASP | 217 | 19 | I | 1.75 | ASP | ASP |
| 198 | 110 | F | 3.76 | ASP | ASP | 218 | 61 | I | 4.15 | ASP | ASP |
| 199 | 116 | F | 2.98 | ASP | ASP | 219 | 65 | I | 2.87 | ASP | ASP |
| 200 | 140 | F | 2.40 | ASP | ASP | 220 | 72 | I | 2.94 | ASP | ASP |
| 201 | 145 | F | 3.45 | ASP | ASP | 221 | 94 | I | 4.08 | ASP | ASP |
| 202 | 154 | F | 3.48 | ASP | ASP | 222 | 113 | I | 2.71 | ASP | ASP |
| 203 | 7 | H | 3.78 | ASP | ASP | 223 | 16 | J | 4.02 | ASP | ASP |
| 204 | 8 | H | 3.95 | ASP | ASP | 224 | 28 | J | 3.80 | ASP | ASP |
| 205 | 16 | H | 3.39 | ASP | ASP | 225 | 31 | J | 2.78 | ASP | ASP |
| 206 | 34 | H | 3.28 | ASP | ASP | 226 | 55 | J | 3.93 | ASP | ASP |
| 207 | 41 | H | 3.44 | ASP | ASP | 227 | 5 | K | 3.49 | ASP | ASP |
| 208 | 53 | H | 3.64 | ASP | ASP | 228 | 22 | K | 3.81 | ASP | ASP |
| 209 | 67 | H | 3.72 | ASP | ASP | 229 | 24 | K | 1.81 | ASP | ASP |
| 210 | 72 | H | 3.67 | ASP | ASP | 230 | 39 | K | 0.00 | ASP | ASP |
| 211 | 86 | H | 3.31 | ASP | ASP | 231 | 53 | K | 3.23 | ASP | ASP |
| 212 | 91 | H | 3.88 | ASP | ASP | 232 | 82 | K | 2.88 | ASP | ASP |
| 213 | 92 | H | 3.02 | ASP | ASP | 233 | 85 | K | 3.43 | ASP | ASP |
| 214 | 94 | H | 2.92 | ASP | ASP | 234 | 44 | L | 2.62 | ASP | ASP |
| 215 | 110 | H | 3.91 | ASP | ASP | 235 | 50 | L | 3.32 | ASP | ASP |
| 216 | 9 | I | 3.19 | ASP | ASP |  |  |  |  |  |  |
