# Supplementary material for: Structural Model of RNA Polymerase II Elongation Complex with Complete Transcription Bubble Reveals NTP Entry Routes
Source: PLoS Comput Biol. 2015 Jul 2;11(7):e1004354. doi: 10.1371/journal.pcbi.1004354 (PMC4489626; doi:10.1371/journal.pcbi.1004354)
Supplement: S2 Table — were obtained by averaging predictions made by the Propka software using 44 MD conformations. Amino acids whose predicted pKa value (Propka) suggests a protonation state that differs from the one used in our MD simulations are highlighted. (DOC) [file pcbi.1004354.s011.doc]

**S2** **Table** **Comparison between protonation states of glutamic acid (GLU) adopted in our MD simulations and those predicted by the Propka software.** <pKa> were obtained by averaging predictions made by the Propka software using 44 MD conformations. Amino acids whose predicted pKa value (Propka) suggests a protonation state that differs from the one used in our MD simulations are highlighted.

| GLU Index | residue ID | chain ID | <pKa> | predicted state | used state | GLU Index | residue ID | chain ID | <pKa> | predicted state | used state |
| --- | --- | --- | --- | --- | --- | --- | --- | --- | --- | --- | --- |
| 1 | 16 | A | 3.92 | GLU | GLU | 50 | 812 | A | 5.18 | GLU | GLU |
| 2 | 25 | A | 3.79 | GLU | GLU | 51 | 822 | A | 5.50 | GLU | GLU |
| 3 | 26 | A | 4.44 | GLU | GLU | 52 | 833 | A | 4.02 | GLU | GLU |
| 4 | 39 | A | 4.99 | GLU | GLU | 53 | 846 | A | 1.94 | GLU | GLU |
| 5 | 43 | A | 4.59 | GLU | GLU | 54 | 870 | A | 4.75 | GLU | GLU |
| 6 | 72 | A | 3.31 | GLU | GLU | 55 | 879 | A | 2.49 | GLU | GLU |
| 7 | 76 | A | 4.46 | GLU | GLU | 56 | 894 | A | 3.71 | GLU | GLU |
| 8 | 104 | A | 3.95 | GLU | GLU | 57 | 914 | A | 4.72 | GLU | GLU |
| 9 | 117 | A | 4.54 | GLU | GLU | 58 | 918 | A | 4.60 | GLU | GLU |
| 10 | 120 | A | 3.72 | GLU | GLU | 59 | 931 | A | 4.04 | GLU | GLU |
| 11 | 149 | A | 3.87 | GLU | GLU | 60 | 932 | A | 6.70 | GLU | GLU |
| 12 | 155 | A | 4.53 | GLU | GLU | 61 | 945 | A | 4.24 | GLU | GLU |
| 13 | 196 | A | 4.83 | GLU | GLU | 62 | 951 | A | 4.26 | GLU | GLU |
| 14 | 198 | A | 4.43 | GLU | GLU | 63 | 995 | A | 3.82 | GLU | GLU |
| 15 | 205 | A | 4.14 | GLU | GLU | 64 | 1005 | A | 4.43 | GLU | GLU |
| 16 | 206 | A | 4.74 | GLU | GLU | 65 | 1034 | A | 3.04 | GLU | GLU |
| 17 | 226 | A | 4.45 | GLU | GLU | 66 | 1050 | A | 4.52 | GLU | GLU |
| 18 | 232 | A | 4.32 | GLU | GLU | 67 | 1062 | A | 2.85 | GLU | GLU |
| 19 | 254 | A | 4.03 | GLU | GLU | 68 | 1074 | A | 5.36 | GLU | GLU |
| 20 | 259 | A | 3.61 | GLU | GLU | 69 | 1103 | A | 4.41 | GLU | GLU |
| 21 | 277 | A | 4.09 | GLU | GLU | 70 | 1121 | A | 4.82 | GLU | GLU |
| 22 | 280 | A | 4.26 | GLU | GLU | 71 | 1129 | A | 4.78 | GLU | GLU |
| 23 | 290 | A | 4.58 | GLU | GLU | 72 | 1139 | A | 3.01 | GLU | GLU |
| 24 | 291 | A | 4.57 | GLU | GLU | 73 | 1151 | A | 4.64 | GLU | GLU |
| 25 | 293 | A | 5.98 | GLU | GLU | 74 | 1165 | A | 3.96 | GLU | GLU |
| 26 | 333 | A | 4.19 | GLU | GLU | 75 | 1167 | A | 4.68 | GLU | GLU |
| 27 | 360 | A | 3.82 | GLU | GLU | 76 | 1168 | A | 4.57 | GLU | GLU |
| 28 | 378 | A | 1.69 | GLU | GLU | 77 | 1179 | A | 3.93 | GLU | GLU |
| 29 | 398 | A | 4.27 | GLU | GLU | 78 | 1180 | A | 4.50 | GLU | GLU |
| 30 | 433 | A | 2.72 | GLU | GLU | 79 | 1182 | A | 4.17 | GLU | GLU |
| 31 | 486 | A | 1.19 | GLU | GLU | 80 | 1196 | A | 3.35 | GLU | GLU |
| 32 | 495 | A | 6.36 | GLU | GLU | 81 | 1214 | A | 4.37 | GLU | GLU |
| 33 | 496 | A | 4.76 | GLU | GLU | 82 | 1230 | A | 4.33 | GLU | GLU |
| 34 | 500 | A | 6.42 | GLU | GLU | 83 | 1234 | A | 4.51 | GLU | GLU |
| 35 | 542 | A | 4.11 | GLU | GLU | 84 | 1251 | A | 3.97 | GLU | GLU |
| 36 | 593 | A | 4.65 | GLU | GLU | 85 | 1253 | A | 3.54 | GLU | GLU |
| 37 | 618 | A | 3.43 | GLU | GLU | 86 | 1255 | A | 3.85 | GLU | GLU |
| 38 | 636 | A | 4.85 | GLU | GLU | 87 | 1256 | A | 4.47 | GLU | GLU |
| 39 | 678 | A | 4.71 | GLU | GLU | 88 | 1264 | A | 5.34 | GLU | GLU |
| 40 | 681 | A | 4.67 | GLU | GLU | 89 | 1269 | A | 3.84 | GLU | GLU |
| 41 | 685 | A | 3.89 | GLU | GLU | 90 | 1277 | A | 4.58 | GLU | GLU |
| 42 | 696 | A | 4.94 | GLU | GLU | 91 | 1280 | A | 4.65 | GLU | GLU |
| 43 | 712 | A | 3.76 | GLU | GLU | 92 | 1297 | A | 4.34 | GLU | GLU |
| 44 | 715 | A | 3.16 | GLU | GLU | 93 | 1301 | A | 4.38 | GLU | GLU |
| 45 | 724 | A | 4.25 | GLU | GLU | 94 | 1303 | A | 3.70 | GLU | GLU |
| 46 | 734 | A | 4.53 | GLU | GLU | 95 | 1307 | A | 4.87 | GLU | GLU |
| 47 | 771 | A | 4.24 | GLU | GLU | 96 | 1315 | A | 4.61 | GLU | GLU |
| 48 | 795 | A | 3.27 | GLU | GLU | 97 | 1337 | A | 5.01 | GLU | GLU |
| 49 | 801 | A | 5.93 | GLU | GLU | 98 | 1342 | A | 0.43 | GLU | GLU |

| GLU Index | residue ID | chain ID | <pKa> | predicted state | used state | GLU Index | residue ID | chain ID | <pKa> | predicted state | used state |
| --- | --- | --- | --- | --- | --- | --- | --- | --- | --- | --- | --- |
| 99 | 1351 | A | 4.60 | GLU | GLU | 148 | 612 | B | 5.08 | GLU | GLU |
| 100 | 1403 | A | 3.17 | GLU | GLU | 149 | 621 | B | 0.16 | GLU | GLU |
| 101 | 1404 | A | 4.70 | GLU | GLU | 150 | 623 | B | 3.01 | GLU | GLU |
| 102 | 1407 | A | 4.15 | GLU | GLU | 151 | 641 | B | 3.91 | GLU | GLU |
| 103 | 1411 | A | 3.17 | GLU | GLU | 152 | 644 | B | 3.84 | GLU | GLU |
| 104 | 1417 | A | 4.81 | GLU | GLU | 153 | 650 | B | 3.98 | GLU | GLU |
| 105 | 1426 | A | 8.33 | GLH | GLU | 154 | 665 | B | 3.80 | GLU | GLU |
| 106 | 21 | B | 4.63 | GLU | GLU | 155 | 670 | B | 4.51 | GLU | GLU |
| 107 | 28 | B | 3.68 | GLU | GLU | 156 | 674 | B | 4.21 | GLU | GLU |
| 108 | 40 | B | 4.64 | GLU | GLU | 157 | 677 | B | 4.68 | GLU | GLU |
| 109 | 65 | B | 4.20 | GLU | GLU | 158 | 678 | B | 4.19 | GLU | GLU |
| 110 | 72 | B | 3.86 | GLU | GLU | 159 | 687 | B | 4.20 | GLU | GLU |
| 111 | 80 | B | 3.54 | GLU | GLU | 160 | 691 | B | 0.00 | GLU | GLU |
| 112 | 89 | B | 4.18 | GLU | GLU | 161 | 696 | B | 3.09 | GLU | GLU |
| 113 | 104 | B | 3.01 | GLU | GLU | 162 | 697 | B | 5.08 | GLU | GLU |
| 114 | 116 | B | 4.25 | GLU | GLU | 163 | 698 | B | 6.17 | GLU | GLU |
| 115 | 138 | B | 3.80 | GLU | GLU | 164 | 699 | B | 5.07 | GLU | GLU |
| 116 | 146 | B | 4.07 | GLU | GLU | 165 | 708 | B | 5.09 | GLU | GLU |
| 117 | 150 | B | 4.55 | GLU | GLU | 166 | 711 | B | 4.42 | GLU | GLU |
| 118 | 154 | B | 4.16 | GLU | GLU | 167 | 714 | B | 4.44 | GLU | GLU |
| 119 | 155 | B | 4.45 | GLU | GLU | 168 | 717 | B | 4.45 | GLU | GLU |
| 120 | 157 | B | 4.54 | GLU | GLU | 169 | 718 | B | 4.19 | GLU | GLU |
| 121 | 161 | B | 3.98 | GLU | GLU | 170 | 742 | B | 3.94 | GLU | GLU |
| 122 | 183 | B | 4.48 | GLU | GLU | 171 | 810 | B | 4.58 | GLU | GLU |
| 123 | 186 | B | 3.32 | GLU | GLU | 172 | 816 | B | 4.20 | GLU | GLU |
| 124 | 194 | B | 5.05 | GLU | GLU | 173 | 836 | B | 4.92 | GLU | GLU |
| 125 | 209 | B | 0.13 | GLU | GLU | 174 | 863 | B | 4.67 | GLU | GLU |
| 126 | 216 | B | 2.23 | GLU | GLU | 175 | 872 | B | 4.73 | GLU | GLU |
| 127 | 239 | B | 3.93 | GLU | GLU | 176 | 875 | B | 3.71 | GLU | GLU |
| 128 | 245 | B | 5.19 | GLU | GLU | 177 | 908 | B | 4.55 | GLU | GLU |
| 129 | 262 | B | 3.59 | GLU | GLU | 178 | 922 | B | 4.59 | GLU | GLU |
| 130 | 296 | B | 5.06 | GLU | GLU | 179 | 923 | B | 4.13 | GLU | GLU |
| 131 | 299 | B | 4.50 | GLU | GLU | 180 | 924 | B | 4.49 | GLU | GLU |
| 132 | 312 | B | 4.29 | GLU | GLU | 181 | 945 | B | 3.85 | GLU | GLU |
| 133 | 319 | B | 4.19 | GLU | GLU | 182 | 997 | B | 4.72 | GLU | GLU |
| 134 | 328 | B | 4.52 | GLU | GLU | 183 | 1004 | B | 4.96 | GLU | GLU |
| 135 | 346 | B | 4.18 | GLU | GLU | 184 | 1028 | B | 3.50 | GLU | GLU |
| 136 | 359 | B | 5.31 | GLU | GLU | 185 | 1041 | B | 3.23 | GLU | GLU |
| 137 | 368 | B | 3.85 | GLU | GLU | 186 | 1053 | B | 4.24 | GLU | GLU |
| 138 | 371 | B | 3.83 | GLU | GLU | 187 | 1061 | B | 4.37 | GLU | GLU |
| 139 | 437 | B | 4.18 | GLU | GLU | 188 | 1070 | B | 6.49 | GLU | GLU |
| 140 | 438 | B | 4.06 | GLU | GLU | 189 | 1120 | B | 4.01 | GLU | GLU |
| 141 | 468 | B | 4.46 | GLU | GLU | 190 | 1132 | B | 2.21 | GLU | GLU |
| 142 | 526 | B | 5.36 | GLU | GLU | 191 | 1134 | B | 6.67 | GLU | GLU |
| 143 | 529 | B | 4.16 | GLU | GLU | 192 | 1149 | B | 5.24 | GLU | GLU |
| 144 | 560 | B | 4.63 | GLU | GLU | 193 | 1153 | B | 5.12 | GLU | GLU |
| 145 | 564 | B | 4.16 | GLU | GLU | 194 | 1181 | B | 4.05 | GLU | GLU |
| 146 | 567 | B | 3.99 | GLU | GLU | 195 | 1206 | B | 4.72 | GLU | GLU |
| 147 | 598 | B | 3.14 | GLU | GLU | 196 | 3 | C | 4.43 | GLU | GLU |

| GLU Index | residue ID | chain ID | <pKa> | predicted state | used state | GLU Index | residue ID | chain ID | <pKa> | predicted state | used state |
| --- | --- | --- | --- | --- | --- | --- | --- | --- | --- | --- | --- |
| 197 | 4 | C | 4.28 | GLU | GLU | 239 | 89 | F | 3.83 | GLU | GLU |
| 198 | 12 | C | 4.46 | GLU | GLU | 240 | 112 | F | 4.29 | GLU | GLU |
| 199 | 40 | C | 3.42 | GLU | GLU | 241 | 114 | F | 3.02 | GLU | GLU |
| 200 | 50 | C | 4.67 | GLU | GLU | 242 | 124 | F | 4.12 | GLU | GLU |
| 201 | 52 | C | 3.63 | GLU | GLU | 243 | 127 | F | 4.01 | GLU | GLU |
| 202 | 61 | C | 5.30 | GLU | GLU | 244 | 144 | F | 3.47 | GLU | GLU |
| 203 | 78 | C | 4.52 | GLU | GLU | 245 | 149 | F | 4.55 | GLU | GLU |
| 204 | 81 | C | 4.35 | GLU | GLU | 246 | 150 | F | 2.53 | GLU | GLU |
| 205 | 89 | C | 4.47 | GLU | GLU | 247 | 14 | H | 4.77 | GLU | GLU |
| 206 | 106 | C | 4.46 | GLU | GLU | 248 | 27 | H | 4.79 | GLU | GLU |
| 207 | 108 | C | 3.86 | GLU | GLU | 249 | 45 | H | 4.12 | GLU | GLU |
| 208 | 138 | C | 4.28 | GLU | GLU | 250 | 66 | H | 3.67 | GLU | GLU |
| 209 | 152 | C | 4.28 | GLU | GLU | 251 | 105 | H | 4.03 | GLU | GLU |
| 210 | 166 | C | 4.53 | GLU | GLU | 252 | 106 | H | 4.23 | GLU | GLU |
| 211 | 177 | C | 5.47 | GLU | GLU | 253 | 126 | H | 3.68 | GLU | GLU |
| 212 | 179 | C | 5.41 | GLU | GLU | 254 | 138 | H | 4.64 | GLU | GLU |
| 213 | 194 | C | 4.35 | GLU | GLU | 255 | 18 | I | 4.48 | GLU | GLU |
| 214 | 200 | C | 3.68 | GLU | GLU | 256 | 21 | I | 4.69 | GLU | GLU |
| 215 | 208 | C | 4.62 | GLU | GLU | 257 | 28 | I | 3.75 | GLU | GLU |
| 216 | 210 | C | 4.29 | GLU | GLU | 258 | 36 | I | 3.78 | GLU | GLU |
| 217 | 215 | C | 4.40 | GLU | GLU | 259 | 37 | I | 4.17 | GLU | GLU |
| 218 | 233 | C | 4.64 | GLU | GLU | 260 | 47 | I | 3.73 | GLU | GLU |
| 219 | 4 | E | 3.90 | GLU | GLU | 261 | 54 | I | 4.56 | GLU | GLU |
| 220 | 6 | E | 3.86 | GLU | GLU | 262 | 74 | I | 3.66 | GLU | GLU |
| 221 | 21 | E | 3.57 | GLU | GLU | 263 | 82 | I | 3.78 | GLU | GLU |
| 222 | 33 | E | 4.61 | GLU | GLU | 264 | 19 | J | 4.59 | GLU | GLU |
| 223 | 34 | E | 4.13 | GLU | GLU | 265 | 27 | J | 3.91 | GLU | GLU |
| 224 | 36 | E | 3.83 | GLU | GLU | 266 | 29 | J | 4.61 | GLU | GLU |
| 225 | 40 | E | 4.47 | GLU | GLU | 267 | 32 | J | 4.66 | GLU | GLU |
| 226 | 66 | E | 4.58 | GLU | GLU | 268 | 58 | J | 4.14 | GLU | GLU |
| 227 | 67 | E | 4.26 | GLU | GLU | 269 | 8 | K | 4.98 | GLU | GLU |
| 228 | 81 | E | 4.93 | GLU | GLU | 270 | 14 | K | 4.56 | GLU | GLU |
| 229 | 85 | E | 4.80 | GLU | GLU | 271 | 16 | K | 3.18 | GLU | GLU |
| 230 | 102 | E | 4.24 | GLU | GLU | 272 | 36 | K | 3.66 | GLU | GLU |
| 231 | 133 | E | 4.33 | GLU | GLU | 273 | 38 | K | 4.19 | GLU | GLU |
| 232 | 137 | E | 4.82 | GLU | GLU | 274 | 49 | K | 4.12 | GLU | GLU |
| 233 | 148 | E | 4.86 | GLU | GLU | 275 | 64 | K | 3.83 | GLU | GLU |
| 234 | 160 | E | 3.68 | GLU | GLU | 276 | 79 | K | 3.96 | GLU | GLU |
| 235 | 163 | E | 3.14 | GLU | GLU | 277 | 106 | K | 4.12 | GLU | GLU |
| 236 | 172 | E | 3.97 | GLU | GLU | 278 | 108 | K | 4.38 | GLU | GLU |
| 237 | 194 | E | 4.12 | GLU | GLU | 279 | 33 | L | 3.82 | GLU | GLU |
| 238 | 203 | E | 5.47 | GLU | GLU | 280 | 68 | L | 5.16 | GLU | GLU |
