# Supplementary material for: Structural Model of RNA Polymerase II Elongation Complex with Complete Transcription Bubble Reveals NTP Entry Routes
Source: PLoS Comput Biol. 2015 Jul 2;11(7):e1004354. doi: 10.1371/journal.pcbi.1004354 (PMC4489626; doi:10.1371/journal.pcbi.1004354)
Supplement: S3 Table — were obtained by averaging predictions made by the Propka software using 44 MD conformations. Amino acids whose predicted pKa value (Propka) suggests a protonation state that differs from the one used in our MD simulations are highlighted. (DOC) [file pcbi.1004354.s012.doc]

**S3** **Table** **Comparison between protonation states of lysine (LYS) adopted in our MD simulations and those predicted by the Propka software.** <pKa> were obtained by averaging predictions made by the Propka software using 44 MD conformations. Amino acids whose predicted pKa value (Propka) suggests a protonation state that differs from the one used in our MD simulations are highlighted.

| LYS Index | residue ID | chain ID | <pKa> | predicted state | used state | LYS Index | residue ID | chain ID | <pKa> | predicted state | used state |
| --- | --- | --- | --- | --- | --- | --- | --- | --- | --- | --- | --- |
| 1 | 15 | A | 9.58 | LYS | LYS | 50 | 705 | A | 10.48 | LYS | LYS |
| 2 | 34 | A | 10.57 | LYS | LYS | 51 | 728 | A | 10.81 | LYS | LYS |
| 3 | 49 | A | 9.69 | LYS | LYS | 52 | 738 | A | 10.46 | LYS | LYS |
| 4 | 66 | A | 10.44 | LYS | LYS | 53 | 744 | A | 10.14 | LYS | LYS |
| 5 | 88 | A | 11.79 | LYS | LYS | 54 | 752 | A | 11.13 | LYS | LYS |
| 6 | 98 | A | 11.08 | LYS | LYS | 55 | 773 | A | 10.58 | LYS | LYS |
| 7 | 100 | A | 9.42 | LYS | LYS | 56 | 789 | A | 10.14 | LYS | LYS |
| 8 | 101 | A | 8.62 | LYS | LYS | 57 | 797 | A | 8.71 | LYS | LYS |
| 9 | 112 | A | 10.69 | LYS | LYS | 58 | 830 | A | 10.47 | LYS | LYS |
| 10 | 129 | A | 10.72 | LYS | LYS | 59 | 843 | A | 10.13 | LYS | LYS |
| 11 | 132 | A | 10.43 | LYS | LYS | 60 | 880 | A | 9.74 | LYS | LYS |
| 12 | 133 | A | 10.02 | LYS | LYS | 61 | 895 | A | 10.91 | LYS | LYS |
| 13 | 143 | A | 9.28 | LYS | LYS | 62 | 924 | A | 10.76 | LYS | LYS |
| 14 | 145 | A | 9.92 | LYS | LYS | 63 | 934 | A | 11.36 | LYS | LYS |
| 15 | 176 | A | 9.54 | LYS | LYS | 64 | 938 | A | 10.34 | LYS | LYS |
| 16 | 180 | A | 10.46 | LYS | LYS | 65 | 941 | A | 10.44 | LYS | LYS |
| 17 | 186 | A | 10.34 | LYS | LYS | 66 | 977 | A | 10.56 | LYS | LYS |
| 18 | 187 | A | 10.15 | LYS | LYS | 67 | 984 | A | 10.48 | LYS | LYS |
| 19 | 212 | A | 10.77 | LYS | LYS | 68 | 991 | A | 10.77 | LYS | LYS |
| 20 | 217 | A | 10.94 | LYS | LYS | 69 | 1003 | A | 10.28 | LYS | LYS |
| 21 | 265 | A | 9.69 | LYS | LYS | 70 | 1039 | A | 11.10 | LYS | LYS |
| 22 | 271 | A | 10.28 | LYS | LYS | 71 | 1092 | A | 10.20 | LYS | LYS |
| 23 | 317 | A | 9.17 | LYS | LYS | 72 | 1093 | A | 10.16 | LYS | LYS |
| 24 | 323 | A | 9.58 | LYS | LYS | 73 | 1102 | A | 10.07 | LYS | LYS |
| 25 | 330 | A | 9.89 | LYS | LYS | 74 | 1109 | A | 10.71 | LYS | LYS |
| 26 | 332 | A | 8.81 | LYS | LYS | 75 | 1112 | A | 10.08 | LYS | LYS |
| 27 | 343 | A | 9.13 | LYS | LYS | 76 | 1132 | A | 10.54 | LYS | LYS |
| 28 | 368 | A | 10.12 | LYS | LYS | 77 | 1144 | A | 10.51 | LYS | LYS |
| 29 | 372 | A | 9.75 | LYS | LYS | 78 | 1205 | A | 10.44 | LYS | LYS |
| 30 | 403 | A | 7.68 | LYS | LYS | 79 | 1217 | A | 10.50 | LYS | LYS |
| 31 | 419 | A | 10.40 | LYS | LYS | 80 | 1221 | A | 10.57 | LYS | LYS |
| 32 | 431 | A | 9.77 | LYS | LYS | 81 | 1235 | A | 11.67 | LYS | LYS |
| 33 | 452 | A | 8.60 | LYS | LYS | 82 | 1246 | A | 11.68 | LYS | LYS |
| 34 | 461 | A | 10.63 | LYS | LYS | 83 | 1261 | A | 10.62 | LYS | LYS |
| 35 | 518 | A | 9.71 | LYS | LYS | 84 | 1262 | A | 10.25 | LYS | LYS |
| 36 | 533 | A | 9.36 | LYS | LYS | 85 | 1286 | A | 10.27 | LYS | LYS |
| 37 | 567 | A | 9.94 | LYS | LYS | 86 | 1290 | A | 10.56 | LYS | LYS |
| 38 | 569 | A | 10.15 | LYS | LYS | 87 | 1300 | A | 10.92 | LYS | LYS |
| 39 | 575 | A | 8.91 | LYS | LYS | 88 | 1350 | A | 9.70 | LYS | LYS |
| 40 | 601 | A | 10.33 | LYS | LYS | 89 | 41 | B | 11.57 | LYS | LYS |
| 41 | 619 | A | 9.58 | LYS | LYS | 90 | 87 | B | 10.61 | LYS | LYS |
| 42 | 620 | A | 10.74 | LYS | LYS | 91 | 94 | B | 10.47 | LYS | LYS |
| 43 | 637 | A | 10.15 | LYS | LYS | 92 | 99 | B | 10.80 | LYS | LYS |
| 44 | 644 | A | 10.82 | LYS | LYS | 93 | 133 | B | 10.35 | LYS | LYS |
| 45 | 651 | A | 9.10 | LYS | LYS | 94 | 134 | B | 10.99 | LYS | LYS |
| 46 | 687 | A | 11.97 | LYS | LYS | 95 | 148 | B | 11.74 | LYS | LYS |
| 47 | 688 | A | 10.95 | LYS | LYS | 96 | 164 | B | 10.09 | LYS | LYS |
| 48 | 689 | A | 10.75 | LYS | LYS | 97 | 177 | B | 10.61 | LYS | LYS |
| 49 | 695 | A | 10.44 | LYS | LYS | 98 | 191 | B | 10.62 | LYS | LYS |

| LYS Index | residue ID | chain ID | <pKa> | predicted state | used state | LYS Index | residue ID | chain ID | <pKa> | predicted state | used state |
| --- | --- | --- | --- | --- | --- | --- | --- | --- | --- | --- | --- |
| 99 | 193 | B | 10.73 | LYS | LYS | 148 | 965 | B | 7.95 | LYS | LYS |
| 100 | 210 | B | 7.50 | LYS | LYS | 149 | 972 | B | 10.89 | LYS | LYS |
| 101 | 227 | B | 10.81 | LYS | LYS | 150 | 979 | B | 6.95 | LYN | LYS |
| 102 | 228 | B | 9.36 | LYS | LYS | 151 | 987 | B | 9.26 | LYS | LYS |
| 103 | 246 | B | 11.30 | LYS | LYS | 152 | 1033 | B | 9.87 | LYS | LYS |
| 104 | 257 | B | 9.82 | LYS | LYS | 153 | 1057 | B | 10.66 | LYS | LYS |
| 105 | 270 | B | 10.12 | LYS | LYS | 154 | 1079 | B | 11.53 | LYS | LYS |
| 106 | 277 | B | 9.37 | LYS | LYS | 155 | 1080 | B | 8.64 | LYS | LYS |
| 107 | 315 | B | 9.97 | LYS | LYS | 156 | 1102 | B | 8.27 | LYS | LYS |
| 108 | 344 | B | 10.39 | LYS | LYS | 157 | 1148 | B | 10.36 | LYS | LYS |
| 109 | 345 | B | 10.67 | LYS | LYS | 158 | 1174 | B | 10.59 | LYS | LYS |
| 110 | 347 | B | 10.50 | LYS | LYS | 159 | 1183 | B | 10.51 | LYS | LYS |
| 111 | 353 | B | 10.82 | LYS | LYS | 160 | 1188 | B | 10.47 | LYS | LYS |
| 112 | 358 | B | 11.75 | LYS | LYS | 161 | 1201 | B | 8.04 | LYS | LYS |
| 113 | 374 | B | 7.97 | LYS | LYS | 162 | 9 | C | 10.68 | LYS | LYS |
| 114 | 393 | B | 10.86 | LYS | LYS | 163 | 15 | C | 10.67 | LYS | LYS |
| 115 | 403 | B | 10.36 | LYS | LYS | 164 | 94 | C | 10.41 | LYS | LYS |
| 116 | 404 | B | 12.10 | LYS | LYS | 165 | 116 | C | 10.86 | LYS | LYS |
| 117 | 418 | B | 10.77 | LYS | LYS | 166 | 137 | C | 10.87 | LYS | LYS |
| 118 | 422 | B | 9.73 | LYS | LYS | 167 | 146 | C | 10.66 | LYS | LYS |
| 119 | 423 | B | 9.77 | LYS | LYS | 168 | 149 | C | 10.70 | LYS | LYS |
| 120 | 426 | B | 9.47 | LYS | LYS | 169 | 154 | C | 11.20 | LYS | LYS |
| 121 | 445 | B | 10.67 | LYS | LYS | 170 | 160 | C | 10.08 | LYS | LYS |
| 122 | 451 | B | 10.14 | LYS | LYS | 171 | 161 | C | 9.00 | LYS | LYS |
| 123 | 458 | B | 9.21 | LYS | LYS | 172 | 165 | C | 12.96 | LYS | LYS |
| 124 | 470 | B | 9.65 | LYS | LYS | 173 | 169 | C | 9.09 | LYS | LYS |
| 125 | 471 | B | 9.39 | LYS | LYS | 174 | 185 | C | 10.88 | LYS | LYS |
| 126 | 507 | B | 9.37 | LYS | LYS | 175 | 187 | C | 10.32 | LYS | LYS |
| 127 | 510 | B | 10.85 | LYS | LYS | 176 | 199 | C | 10.55 | LYS | LYS |
| 128 | 537 | B | 8.79 | LYS | LYS | 177 | 205 | C | 11.23 | LYS | LYS |
| 129 | 606 | B | 10.47 | LYS | LYS | 178 | 222 | C | 11.03 | LYS | LYS |
| 130 | 622 | B | 10.09 | LYS | LYS | 179 | 253 | C | 11.16 | LYS | LYS |
| 131 | 625 | B | 12.87 | LYS | LYS | 180 | 254 | C | 13.35 | LYS | LYS |
| 132 | 649 | B | 9.76 | LYS | LYS | 181 | 20 | E | 8.24 | LYS | LYS |
| 133 | 652 | B | 10.86 | LYS | LYS | 182 | 24 | E | 10.35 | LYS | LYS |
| 134 | 655 | B | 11.21 | LYS | LYS | 183 | 43 | E | 11.00 | LYS | LYS |
| 135 | 660 | B | 10.84 | LYS | LYS | 184 | 45 | E | 11.66 | LYS | LYS |
| 136 | 727 | B | 10.87 | LYS | LYS | 185 | 56 | E | 10.67 | LYS | LYS |
| 137 | 775 | B | 7.84 | LYS | LYS | 186 | 71 | E | 10.88 | LYS | LYS |
| 138 | 801 | B | 9.62 | LYS | LYS | 187 | 91 | E | 10.21 | LYS | LYS |
| 139 | 813 | B | 10.10 | LYS | LYS | 188 | 94 | E | 10.19 | LYS | LYS |
| 140 | 864 | B | 10.32 | LYS | LYS | 189 | 103 | E | 10.54 | LYS | LYS |
| 141 | 865 | B | 10.56 | LYS | LYS | 190 | 122 | E | 9.93 | LYS | LYS |
| 142 | 876 | B | 10.35 | LYS | LYS | 191 | 152 | E | 10.33 | LYS | LYS |
| 143 | 886 | B | 9.96 | LYS | LYS | 192 | 161 | E | 10.50 | LYS | LYS |
| 144 | 892 | B | 10.94 | LYS | LYS | 193 | 166 | E | 10.32 | LYS | LYS |
| 145 | 914 | B | 11.22 | LYS | LYS | 194 | 171 | E | 10.40 | LYS | LYS |
| 146 | 934 | B | 10.25 | LYS | LYS | 195 | 191 | E | 10.18 | LYS | LYS |
| 147 | 962 | B | 10.20 | LYS | LYS | 196 | 197 | E | 9.72 | LYS | LYS |

| LYS Index | residue ID | chain ID | <pKa> | predicted state | used state | LYS Index | residue ID | chain ID | <pKa> | predicted state | used state |
| --- | --- | --- | --- | --- | --- | --- | --- | --- | --- | --- | --- |
| 197 | 201 | E | 9.85 | LYS | LYS | 216 | 42 | J | 10.13 | LYS | LYS |
| 198 | 72 | F | 10.63 | LYS | LYS | 217 | 59 | J | 10.74 | LYS | LYS |
| 199 | 76 | F | 10.83 | LYS | LYS | 218 | 18 | K | 9.91 | LYS | LYS |
| 200 | 87 | F | 11.71 | LYS | LYS | 219 | 20 | K | 10.61 | LYS | LYS |
| 201 | 123 | F | 11.43 | LYS | LYS | 220 | 26 | K | 11.11 | LYS | LYS |
| 202 | 128 | F | 10.17 | LYS | LYS | 221 | 37 | K | 11.35 | LYS | LYS |
| 203 | 129 | F | 10.77 | LYS | LYS | 222 | 55 | K | 10.67 | LYS | LYS |
| 204 | 22 | H | 10.95 | LYS | LYS | 223 | 62 | K | 8.63 | LYS | LYS |
| 205 | 37 | H | 11.14 | LYS | LYS | 224 | 72 | K | 10.36 | LYS | LYS |
| 206 | 103 | H | 10.52 | LYS | LYS | 225 | 84 | K | 11.74 | LYS | LYS |
| 207 | 109 | H | 10.87 | LYS | LYS | 226 | 88 | K | 11.13 | LYS | LYS |
| 208 | 136 | H | 10.83 | LYS | LYS | 227 | 97 | K | 11.13 | LYS | LYS |
| 209 | 20 | I | 11.69 | LYS | LYS | 228 | 102 | K | 11.06 | LYS | LYS |
| 210 | 77 | I | 10.78 | LYS | LYS | 229 | 28 | L | 12.40 | LYS | LYS |
| 211 | 93 | I | 10.50 | LYS | LYS | 230 | 37 | L | 10.49 | LYS | LYS |
| 212 | 115 | I | 10.43 | LYS | LYS | 231 | 49 | L | 10.41 | LYS | LYS |
| 213 | 117 | I | 10.43 | LYS | LYS | 232 | 58 | L | 9.86 | LYS | LYS |
| 214 | 12 | J | 11.35 | LYS | LYS | 233 | 62 | L | 10.77 | LYS | LYS |
| 215 | 17 | J | 11.07 | LYS | LYS |  |  |  |  |  |  |
