# Supplementary material for: Structural Model of RNA Polymerase II Elongation Complex with Complete Transcription Bubble Reveals NTP Entry Routes
Source: PLoS Comput Biol. 2015 Jul 2;11(7):e1004354. doi: 10.1371/journal.pcbi.1004354 (PMC4489626; doi:10.1371/journal.pcbi.1004354)
Supplement: S4 Table — were obtained by averaging predictions made by the Propka software using 44 MD conformations. (DOC) [file pcbi.1004354.s013.doc]

**S4** **Table** **Comparison between protonation states of arginine (ARG) adopted in our MD simulations and those predicted by the Propka software.** <pKa> were obtained by averaging predictions made by the Propka software using 44 MD conformations.

| ARG Index | residue ID | chain ID | <pKa> | predicted state | used state | ARG Index | residue ID | chain ID | <pKa> | predicted state | used state |
| --- | --- | --- | --- | --- | --- | --- | --- | --- | --- | --- | --- |
| 1 | 12 | A | 12.76 | ARG | ARG | 50 | 896 | A | 13.85 | ARG | ARG |
| 2 | 28 | A | 13.94 | ARG | ARG | 51 | 898 | A | 13.92 | ARG | ARG |
| 3 | 36 | A | 13.23 | ARG | ARG | 52 | 940 | A | 11.56 | ARG | ARG |
| 4 | 47 | A | 12.96 | ARG | ARG | 53 | 944 | A | 12.38 | ARG | ARG |
| 5 | 57 | A | 12.52 | ARG | ARG | 54 | 961 | A | 11.20 | ARG | ARG |
| 6 | 63 | A | 12.50 | ARG | ARG | 55 | 962 | A | 14.00 | ARG | ARG |
| 7 | 123 | A | 13.07 | ARG | ARG | 56 | 1001 | A | 11.64 | ARG | ARG |
| 8 | 134 | A | 12.17 | ARG | ARG | 57 | 1012 | A | 13.24 | ARG | ARG |
| 9 | 164 | A | 13.04 | ARG | ARG | 58 | 1023 | A | 13.27 | ARG | ARG |
| 10 | 175 | A | 12.57 | ARG | ARG | 59 | 1025 | A | 12.12 | ARG | ARG |
| 11 | 189 | A | 11.68 | ARG | ARG | 60 | 1029 | A | 11.47 | ARG | ARG |
| 12 | 200 | A | 13.23 | ARG | ARG | 61 | 1030 | A | 14.00 | ARG | ARG |
| 13 | 230 | A | 13.43 | ARG | ARG | 62 | 1036 | A | 11.25 | ARG | ARG |
| 14 | 247 | A | 12.18 | ARG | ARG | 63 | 1055 | A | 13.61 | ARG | ARG |
| 15 | 257 | A | 13.11 | ARG | ARG | 64 | 1100 | A | 13.53 | ARG | ARG |
| 16 | 320 | A | 11.51 | ARG | ARG | 65 | 1135 | A | 14.00 | ARG | ARG |
| 17 | 326 | A | 13.23 | ARG | ARG | 66 | 1159 | A | 13.65 | ARG | ARG |
| 18 | 328 | A | 13.10 | ARG | ARG | 67 | 1194 | A | 14.00 | ARG | ARG |
| 19 | 335 | A | 13.09 | ARG | ARG | 68 | 1199 | A | 13.07 | ARG | ARG |
| 20 | 337 | A | 11.69 | ARG | ARG | 69 | 1215 | A | 12.02 | ARG | ARG |
| 21 | 344 | A | 10.95 | ARG | ARG | 70 | 1239 | A | 11.53 | ARG | ARG |
| 22 | 350 | A | 12.09 | ARG | ARG | 71 | 1241 | A | 11.72 | ARG | ARG |
| 23 | 387 | A | 13.93 | ARG | ARG | 72 | 1244 | A | 12.92 | ARG | ARG |
| 24 | 393 | A | 12.41 | ARG | ARG | 73 | 1274 | A | 11.56 | ARG | ARG |
| 25 | 407 | A | 12.89 | ARG | ARG | 74 | 1281 | A | 13.38 | ARG | ARG |
| 26 | 412 | A | 12.61 | ARG | ARG | 75 | 1289 | A | 12.79 | ARG | ARG |
| 27 | 416 | A | 13.00 | ARG | ARG | 76 | 1326 | A | 13.86 | ARG | ARG |
| 28 | 420 | A | 12.39 | ARG | ARG | 77 | 1345 | A | 13.92 | ARG | ARG |
| 29 | 434 | A | 13.53 | ARG | ARG | 78 | 1366 | A | 13.81 | ARG | ARG |
| 30 | 446 | A | 11.77 | ARG | ARG | 79 | 1386 | A | 12.50 | ARG | ARG |
| 31 | 459 | A | 13.44 | ARG | ARG | 80 | 1391 | A | 12.02 | ARG | ARG |
| 32 | 469 | A | 12.22 | ARG | ARG | 81 | 1399 | A | 12.76 | ARG | ARG |
| 33 | 498 | A | 14.00 | ARG | ARG | 82 | 1422 | A | 12.65 | ARG | ARG |
| 34 | 532 | A | 11.71 | ARG | ARG | 83 | 39 | B | 13.29 | ARG | ARG |
| 35 | 537 | A | 14.00 | ARG | ARG | 84 | 86 | B | 13.43 | ARG | ARG |
| 36 | 590 | A | 12.06 | ARG | ARG | 85 | 118 | B | 14.00 | ARG | ARG |
| 37 | 635 | A | 10.66 | ARG | ARG | 86 | 120 | B | 12.44 | ARG | ARG |
| 38 | 677 | A | 13.38 | ARG | ARG | 87 | 135 | B | 13.78 | ARG | ARG |
| 39 | 711 | A | 12.73 | ARG | ARG | 88 | 145 | B | 13.73 | ARG | ARG |
| 40 | 720 | A | 13.29 | ARG | ARG | 89 | 169 | B | 11.32 | ARG | ARG |
| 41 | 726 | A | 11.89 | ARG | ARG | 90 | 175 | B | 12.10 | ARG | ARG |
| 42 | 731 | A | 13.28 | ARG | ARG | 91 | 217 | B | 12.71 | ARG | ARG |
| 43 | 774 | A | 11.56 | ARG | ARG | 92 | 241 | B | 12.25 | ARG | ARG |
| 44 | 782 | A | 11.01 | ARG | ARG | 93 | 249 | B | 12.68 | ARG | ARG |
| 45 | 806 | A | 10.47 | ARG | ARG | 94 | 261 | B | 12.64 | ARG | ARG |
| 46 | 821 | A | 10.44 | ARG | ARG | 95 | 267 | B | 11.29 | ARG | ARG |
| 47 | 839 | A | 14.00 | ARG | ARG | 96 | 287 | B | 11.55 | ARG | ARG |
| 48 | 840 | A | 11.22 | ARG | ARG | 97 | 327 | B | 12.80 | ARG | ARG |
| 49 | 857 | A | 12.28 | ARG | ARG | 98 | 336 | B | 11.46 | ARG | ARG |

| ARG Index | residue ID | chain ID | <pKa> | predicted state | used state | ARG Index | residue ID | chain ID | <pKa> | predicted state | used state |
| --- | --- | --- | --- | --- | --- | --- | --- | --- | --- | --- | --- |
| 99 | 337 | B | 12.09 | ARG | ARG | 148 | 1094 | B | 12.00 | ARG | ARG |
| 100 | 348 | B | 13.12 | ARG | ARG | 149 | 1096 | B | 11.34 | ARG | ARG |
| 101 | 373 | B | 12.12 | ARG | ARG | 150 | 1106 | B | 12.48 | ARG | ARG |
| 102 | 384 | B | 14.00 | ARG | ARG | 151 | 1108 | B | 13.23 | ARG | ARG |
| 103 | 392 | B | 12.93 | ARG | ARG | 152 | 1116 | B | 11.67 | ARG | ARG |
| 104 | 398 | B | 12.69 | ARG | ARG | 153 | 1122 | B | 11.26 | ARG | ARG |
| 105 | 405 | B | 12.79 | ARG | ARG | 154 | 1124 | B | 11.38 | ARG | ARG |
| 106 | 430 | B | 12.04 | ARG | ARG | 155 | 1129 | B | 11.73 | ARG | ARG |
| 107 | 434 | B | 12.01 | ARG | ARG | 156 | 1135 | B | 14.00 | ARG | ARG |
| 108 | 476 | B | 11.23 | ARG | ARG | 157 | 1150 | B | 11.50 | ARG | ARG |
| 109 | 485 | B | 14.00 | ARG | ARG | 158 | 1159 | B | 12.35 | ARG | ARG |
| 110 | 496 | B | 10.25 | ARG | ARG | 159 | 1215 | B | 12.34 | ARG | ARG |
| 111 | 497 | B | 10.26 | ARG | ARG | 160 | 1220 | B | 12.32 | ARG | ARG |
| 112 | 504 | B | 11.66 | ARG | ARG | 161 | 1222 | B | 13.26 | ARG | ARG |
| 113 | 512 | B | 12.14 | ARG | ARG | 162 | 11 | C | 13.21 | ARG | ARG |
| 114 | 579 | B | 14.00 | ARG | ARG | 163 | 34 | C | 9.43 | ARG | ARG |
| 115 | 591 | B | 13.29 | ARG | ARG | 164 | 35 | C | 14.00 | ARG | ARG |
| 116 | 595 | B | 13.04 | ARG | ARG | 165 | 66 | C | 11.07 | ARG | ARG |
| 117 | 601 | B | 12.37 | ARG | ARG | 166 | 84 | C | 12.72 | ARG | ARG |
| 118 | 604 | B | 14.00 | ARG | ARG | 167 | 127 | C | 12.91 | ARG | ARG |
| 119 | 605 | B | 12.53 | ARG | ARG | 168 | 148 | C | 12.32 | ARG | ARG |
| 120 | 617 | B | 11.40 | ARG | ARG | 169 | 246 | C | 12.47 | ARG | ARG |
| 121 | 620 | B | 13.34 | ARG | ARG | 170 | 7 | E | 13.87 | ARG | ARG |
| 122 | 632 | B | 12.57 | ARG | ARG | 171 | 11 | E | 11.96 | ARG | ARG |
| 123 | 635 | B | 14.00 | ARG | ARG | 172 | 14 | E | 11.29 | ARG | ARG |
| 124 | 654 | B | 13.53 | ARG | ARG | 173 | 17 | E | 12.91 | ARG | ARG |
| 125 | 728 | B | 12.26 | ARG | ARG | 174 | 26 | E | 12.64 | ARG | ARG |
| 126 | 730 | B | 12.91 | ARG | ARG | 175 | 52 | E | 13.54 | ARG | ARG |
| 127 | 766 | B | 10.73 | ARG | ARG | 176 | 55 | E | 12.92 | ARG | ARG |
| 128 | 788 | B | 14.00 | ARG | ARG | 177 | 155 | E | 13.02 | ARG | ARG |
| 129 | 807 | B | 14.00 | ARG | ARG | 178 | 162 | E | 13.77 | ARG | ARG |
| 130 | 815 | B | 14.00 | ARG | ARG | 179 | 167 | E | 13.22 | ARG | ARG |
| 131 | 848 | B | 9.87 | ARG | ARG | 180 | 169 | E | 12.73 | ARG | ARG |
| 132 | 852 | B | 14.00 | ARG | ARG | 181 | 177 | E | 11.75 | ARG | ARG |
| 133 | 857 | B | 13.17 | ARG | ARG | 182 | 180 | E | 13.38 | ARG | ARG |
| 134 | 879 | B | 12.37 | ARG | ARG | 183 | 192 | E | 12.50 | ARG | ARG |
| 135 | 884 | B | 11.63 | ARG | ARG | 184 | 200 | E | 14.00 | ARG | ARG |
| 136 | 904 | B | 12.12 | ARG | ARG | 185 | 207 | E | 13.07 | ARG | ARG |
| 137 | 928 | B | 10.70 | ARG | ARG | 186 | 212 | E | 12.08 | ARG | ARG |
| 138 | 935 | B | 11.63 | ARG | ARG | 187 | 79 | F | 13.76 | ARG | ARG |
| 139 | 942 | B | 11.08 | ARG | ARG | 188 | 90 | F | 11.75 | ARG | ARG |
| 140 | 967 | B | 11.99 | ARG | ARG | 189 | 92 | F | 12.47 | ARG | ARG |
| 141 | 969 | B | 14.00 | ARG | ARG | 190 | 97 | F | 12.71 | ARG | ARG |
| 142 | 983 | B | 12.46 | ARG | ARG | 191 | 119 | F | 12.67 | ARG | ARG |
| 143 | 995 | B | 13.47 | ARG | ARG | 192 | 135 | F | 13.25 | ARG | ARG |
| 144 | 996 | B | 12.18 | ARG | ARG | 193 | 136 | F | 14.00 | ARG | ARG |
| 145 | 1020 | B | 14.00 | ARG | ARG | 194 | 19 | H | 12.59 | ARG | ARG |
| 146 | 1060 | B | 12.87 | ARG | ARG | 195 | 25 | H | 12.75 | ARG | ARG |
| 147 | 1067 | B | 13.74 | ARG | ARG | 196 | 77 | H | 13.07 | ARG | ARG |

| ARG Index | residue ID | chain ID | <pKa> | predicted state | used state | ARG Index | residue ID | chain ID | <pKa> | predicted state | used state |
| --- | --- | --- | --- | --- | --- | --- | --- | --- | --- | --- | --- |
| 197 | 80 | H | 12.21 | ARG | ARG | 215 | 6 | J | 10.13 | ARG | ARG |
| 198 | 87 | H | 12.62 | ARG | ARG | 216 | 38 | J | 12.49 | ARG | ARG |
| 199 | 124 | H | 14.00 | ARG | ARG | 217 | 43 | J | 13.11 | ARG | ARG |
| 200 | 130 | H | 12.24 | ARG | ARG | 218 | 47 | J | 11.52 | ARG | ARG |
| 201 | 145 | H | 13.65 | ARG | ARG | 219 | 48 | J | 11.01 | ARG | ARG |
| 202 | 146 | H | 13.32 | ARG | ARG | 220 | 62 | J | 13.66 | ARG | ARG |
| 203 | 5 | I | 12.31 | ARG | ARG | 221 | 6 | K | 14.00 | ARG | ARG |
| 204 | 8 | I | 13.25 | ARG | ARG | 222 | 47 | K | 12.86 | ARG | ARG |
| 205 | 17 | I | 13.20 | ARG | ARG | 223 | 54 | K | 12.42 | ARG | ARG |
| 206 | 24 | I | 13.58 | ARG | ARG | 224 | 70 | K | 13.36 | ARG | ARG |
| 207 | 30 | I | 13.77 | ARG | ARG | 225 | 74 | K | 12.80 | ARG | ARG |
| 208 | 45 | I | 14.00 | ARG | ARG | 226 | 42 | L | 12.72 | ARG | ARG |
| 209 | 70 | I | 12.23 | ARG | ARG | 227 | 47 | L | 13.34 | ARG | ARG |
| 210 | 73 | I | 12.20 | ARG | ARG | 228 | 54 | L | 12.39 | ARG | ARG |
| 211 | 81 | I | 13.57 | ARG | ARG | 229 | 60 | L | 12.18 | ARG | ARG |
| 212 | 91 | I | 13.83 | ARG | ARG | 230 | 63 | L | 12.90 | ARG | ARG |
| 213 | 92 | I | 13.38 | ARG | ARG | 231 | 70 | L | 14.00 | ARG | ARG |
| 214 | 118 | I | 13.53 | ARG | ARG |  |  |  |  |  |  |
