# Supplementary material for: Structural Model of RNA Polymerase II Elongation Complex with Complete Transcription Bubble Reveals NTP Entry Routes
Source: PLoS Comput Biol. 2015 Jul 2;11(7):e1004354. doi: 10.1371/journal.pcbi.1004354 (PMC4489626; doi:10.1371/journal.pcbi.1004354)
Supplement: S5 Table — were obtained by averaging predictions made by the Propka software using 44 MD conformations. Amino acids whose predicted pKa value (Propka) suggests a protonation state that differs from the one used in our MD simulations are highlighted. (DOC) [file pcbi.1004354.s014.doc]

**S5** **Table** **Comparison between protonation states of histidine (HIS) adopted in our MD simulations and those predicted by the Propka software.** <pKa> were obtained by averaging predictions made by the Propka software using 44 MD conformations. Amino acids whose predicted pKa value (Propka) suggests a protonation state that differs from the one used in our MD simulations are highlighted.

| HIS Index | residue ID | chain ID | <pKa> | predicted state | used state | HIS Index | residue ID | chain ID | <pKa> | predicted state | used state |
| --- | --- | --- | --- | --- | --- | --- | --- | --- | --- | --- | --- |
| 1 | 80 | A | 4.66 | HIS | HIS | 43 | 515 | B | 5.13 | HIS | HIS |
| 2 | 83 | A | 5.17 | HIS | HIS | 44 | 518 | B | 1.04 | HIS | HIS |
| 3 | 92 | A | 3.83 | HIS | HIS | 45 | 572 | B | 6.28 | HIS | HIS |
| 4 | 109 | A | 6.30 | HIS | HIS | 46 | 587 | B | 4.58 | HIS | HIS |
| 5 | 118 | A | 6.32 | HIS | HIS | 47 | 590 | B | 5.38 | HIS | HIS |
| 6 | 213 | A | 6.10 | HIS | HIS | 48 | 648 | B | 6.65 | HIS | HIS |
| 7 | 281 | A | 6.47 | HIS | HIS | 49 | 657 | B | 5.57 | HIS | HIS |
| 8 | 286 | A | 6.78 | HIS | HIS | 50 | 733 | B | 5.69 | HIS | HIS |
| 9 | 287 | A | 6.28 | HIS | HIS | 51 | 734 | B | 6.62 | HIS | HIS |
| 10 | 299 | A | 5.19 | HIS | HIS | 52 | 740 | B | 5.36 | HIS | HIS |
| 11 | 399 | A | 5.28 | HIS | HIS | 53 | 744 | B | 3.65 | HIS | HIS |
| 12 | 435 | A | 3.64 | HIS | HIS | 54 | 761 | B | 4.81 | HIS | HIS |
| 13 | 451 | A | 4.17 | HIS | HIS | 55 | 887 | B | 5.99 | HIS | HIS |
| 14 | 458 | A | 3.68 | HIS | HIS | 56 | 932 | B | 6.02 | HIS | HIS |
| 15 | 490 | A | 2.85 | HIS | HIS | 57 | 984 | B | 4.39 | HIS | HIS |
| 16 | 587 | A | 4.79 | HIS | HIS | 58 | 1015 | B | 4.23 | HIS | HIS |
| 17 | 631 | A | 4.52 | HIS | HIS | 59 | 1025 | B | 3.11 | HIS | HIS |
| 18 | 659 | A | 3.67 | HIS | HIS | 60 | 1062 | B | 5.65 | HIS | HIS |
| 19 | 706 | A | 6.25 | HIS | HIS | 61 | 1076 | B | 4.10 | HIS | HIS |
| 20 | 786 | A | 3.53 | HIS | HIS | 62 | 1097 | B | 0.34 | HIS | HIS |
| 21 | 816 | A | 4.22 | HIS | HIS | 63 | 1104 | B | 4.50 | HIS | HIS |
| 22 | 851 | A | 4.08 | HIS | HIS | 64 | 1141 | B | 2.95 | HIS | HIS |
| 23 | 877 | A | 2.79 | HIS | HIS | 65 | 1161 | B | 4.52 | HIS | HIS |
| 24 | 906 | A | 5.99 | HIS | HIS | 66 | 1177 | B | 6.07 | HIS | HIS |
| 25 | 972 | A | 6.28 | HIS | HIS | 67 | 1195 | B | 5.34 | HIS | HIS |
| 26 | 975 | A | 5.07 | HIS | HIS | 68 | 65 | C | 3.63 | HIS | HIS |
| 27 | 1059 | A | 4.16 | HIS | HIS | 69 | 91 | C | 7.02 | HIP | HIS |
| 28 | 1085 | A | 5.91 | HIS | HIP | 70 | 131 | C | 5.89 | HIS | HIS |
| 29 | 1124 | A | 6.28 | HIS | HIS | 71 | 167 | C | 5.15 | HIS | HIS |
| 30 | 1140 | A | 5.82 | HIS | HIS | 72 | 188 | C | 4.64 | HIS | HIS |
| 31 | 1173 | A | 5.17 | HIS | HIS | 73 | 99 | E | 5.76 | HIS | HIS |
| 32 | 1258 | A | 6.23 | HIS | HIS | 74 | 146 | E | 7.00 | HIS | HIS |
| 33 | 1367 | A | 3.76 | HIS | HIS | 75 | 147 | E | 4.80 | HIS | HIS |
| 34 | 1387 | A | 4.15 | HIS | HIS | 76 | 153 | E | 4.58 | HIS | HIS |
| 35 | 77 | B | 5.26 | HIS | HIS | 77 | 46 | I | 4.76 | HIS | HIS |
| 36 | 110 | B | 6.24 | HIS | HIS | 78 | 79 | I | 6.63 | HIS | HIS |
| 37 | 236 | B | 4.26 | HIS | HIS | 79 | 108 | I | 6.36 | HIS | HIS |
| 38 | 300 | B | 4.37 | HIS | HIS | 80 | 53 | J | 4.92 | HIS | HIS |
| 39 | 363 | B | 4.16 | HIS | HIS | 81 | 40 | K | 2.52 | HIS | HIS |
| 40 | 400 | B | 3.80 | HIS | HIS | 82 | 65 | K | 4.65 | HIS | HIS |
| 41 | 440 | B | 6.29 | HIS | HIS | 83 | 53 | L | 5.59 | HIS | HIS |
| 42 | 494 | B | 1.82 | HIS | HIS |  |  |  |  |  |  |
