# Supplementary material for: Structural Model of RNA Polymerase II Elongation Complex with Complete Transcription Bubble Reveals NTP Entry Routes
Source: PLoS Comput Biol. 2015 Jul 2;11(7):e1004354. doi: 10.1371/journal.pcbi.1004354 (PMC4489626; doi:10.1371/journal.pcbi.1004354)
Supplement: S6 Table — The table presents the distance to the NTP diffusion pathways for the six amino acids whose predicted pKa value (Propka) suggests a protonation state that differs from the one used in our MD simulations. The distances are the average minimum distance from the 44 MD conformations used for Propka predictions. (DOC) [file pcbi.1004354.s015.doc]

**S6 Table** **Distances between the amino acids and the main/secondary channel.** The table presents the distance to the NTP diffusion pathways for the six amino acids whose predicted pKa value (Propka) suggests a protonation state that differs from the one used in our MD simulations. The distances are the average minimum distance from the 44 MD conformations used for Propka predictions.

| Residue | Distance (Å) | |
| --- | --- | --- |
|  | to main channel | to secondary channel |
| GLU_A1426 | 31.9 | 32.8 |
| LYS_B979 | 34.9 | 13.4 |
| HIS_A1085 | 33.5 | 7.6 |
| HIS_B734 | 41.5 | 26.9 |
| HIS_C91 | 71.6 | 55.7 |
| HIS_E146 | 52.2 | 26.7 |
