# Supplementary material for: Structural Model of RNA Polymerase II Elongation Complex with Complete Transcription Bubble Reveals NTP Entry Routes
Source: PLoS Comput Biol. 2015 Jul 2;11(7):e1004354. doi: 10.1371/journal.pcbi.1004354 (PMC4489626; doi:10.1371/journal.pcbi.1004354)
Supplement: S7 Table — (DOC) [file pcbi.1004354.s016.doc]

**S7 Table** **Partial charges of (UTP-Mg)^2-^ group**

| atom name | partial charge |  | atom name | partial charge |
| --- | --- | --- | --- | --- |
| PA | 0.80 |  | C6 | 0.07 |
| O1A | -0.66 |  | C5 | -0.61 |
| O2A | -0.66 |  | C4 | 0.93 |
| O3A | -0.39 |  | N3 | -0.74 |
| PB | 1.21 |  | C2 | 0.83 |
| O1B | -0.76 |  | O2 | -0.65 |
| O2B | -0.76 |  | H3 | 0.38 |
| O3B | -0.48 |  | O4 | -0.67 |
| PG | 1.27 |  | H5 | 0.22 |
| O1G | -0.82 |  | H6 | 0.23 |
| O2G | -0.82 |  | H1' | 0.12 |
| O3G | -0.82 |  | C2' | 0.30 |
| O5' | -0.40 |  | O2' | -0.78 |
| C5' | 0.24 |  | HO'2 | 0.50 |
| H5'1 | 0.04 |  | H2'1 | -0.02 |
| H5'2 | 0.04 |  | C3' | 0.46 |
| C4' | -0.05 |  | H3' | -0.12 |
| H4' | 0.14 |  | O3' | -0.84 |
| O4' | -0.52 |  | H3T | 0.54 |
| C1' | 0.28 |  | MG | 1.20 |
| N1 | -0.22 |  |  |  |
