# Supplementary material for: Structural Model of RNA Polymerase II Elongation Complex with Complete Transcription Bubble Reveals NTP Entry Routes
Source: PLoS Comput Biol. 2015 Jul 2;11(7):e1004354. doi: 10.1371/journal.pcbi.1004354 (PMC4489626; doi:10.1371/journal.pcbi.1004354)
Supplement: S8 Table — (DOC) [file pcbi.1004354.s017.doc]

**S8 Table Partial charges of (ATP-Mg)^2-^ group**

| atom name | partial charge |  | atom name | partial charge |
| --- | --- | --- | --- | --- |
| PA | 0.79 |  | HO'2 | 0.51 |
| O1A | -0.66 |  | H2'1 | -0.02 |
| O2A | -0.66 |  | H3' | -0.08 |
| O3A | -0.39 |  | H4' | 0.13 |
| PB | 1.22 |  | O4' | -0.58 |
| O1B | -0.77 |  | C1' | 0.49 |
| O2B | -0.77 |  | H1' | 0.07 |
| O3B | -0.48 |  | N9 | -0.28 |
| PG | 1.27 |  | C8 | 0.26 |
| O1G | -0.82 |  | H8 | 0.17 |
| O2G | -0.82 |  | N7 | -0.62 |
| O3G | -0.82 |  | C5 | -0.04 |
| O5' | -0.40 |  | C6 | 0.77 |
| C5' | 0.25 |  | N6 | -0.94 |
| H5'1 | 0.03 |  | H61 | 0.40 |
| H5'2 | 0.03 |  | H62 | 0.40 |
| C4' | 0.00 |  | N1 | -0.84 |
| C3' | 0.33 |  | C2 | 0.63 |
| O3' | -0.83 |  | H2 | 0.04 |
| HO'3 | 0.55 |  | N3 | -0.80 |
| C2' | 0.33 |  | C4 | 0.55 |
| O2' | -0.80 |  | MG | 1.20 |
